# Identification of sample-specific regulations using integrative network level analysis

**DOI:** 10.1186/s12885-015-1265-2

**Published:** 2015-04-28

**Authors:** Chengyu Liu, Riku Louhimo, Marko Laakso, Rainer Lehtonen, Sampsa Hautaniemi

**Affiliations:** Research Programs Unit, Genome-Scale Biology Research Program and Institute of Biomedicine, University of Helsinki, Haartmaninkatu 8, Helsinki, FI-00014 Finland

**Keywords:** Sample-specific, Network, DERA, Personalized, Pathway

## Abstract

**Background:**

Histologically similar tumors even from the same anatomical position may still show high variability at molecular level hindering analysis of genome-wide data. Leveling the analysis to a gene regulatory network instead of focusing on single genes has been suggested to overcome the heterogeneity issue although the majority of the network methods require large datasets. Network methods that are able to function at a single sample level are needed to overcome the heterogeneity and sample size issues.

**Methods:**

We present a novel network method, Differentially Expressed Regulation Analysis (DERA) that integrates expression data to biological network information at a single sample level. The sample-specific networks are subsequently used to discover samples with similar molecular functions by identification of regulations that are shared between samples or are specific for a subgroup.

**Results:**

We applied DERA to identify key regulations in triple negative breast cancer (TNBC), which is characterized by lack of estrogen receptor, progesterone receptor and HER2 expression and has poorer prognosis than the other breast cancer subtypes. DERA identified 110 core regulations consisting of 28 disconnected subnetworks for TNBC. These subnetworks are related to oncogenic activity, proliferation, cancer survival, invasiveness and metastasis. Our analysis further revealed 31 regulations specific for TNBC as compared to the other breast cancer subtypes and thus form a basis for understanding TNBC. We also applied DERA to high-grade serous ovarian cancer (HGS-OvCa) data and identified several common regulations between HGS-OvCa and TNBC. The performance of DERA was compared to two pathway analysis methods GSEA and SPIA and our results shows better reproducibility and higher sensitivity in a small sample set.

**Conclusions:**

We present a novel method called DERA to identify subnetworks that are similarly active for a group of samples. DERA was applied to breast cancer and ovarian cancer data showing our method is able to identify reliable and potentially important regulations with high reproducibility. R package is available at http://csbi.ltdk.helsinki.fi/pub/czliu/DERA/.

**Electronic supplementary material:**

The online version of this article (doi:10.1186/s12885-015-1265-2) contains supplementary material, which is available to authorized users.

## Background

Novel measurement technologies, such as microarrays and deep sequencing, provide quantitative genome-scale data from diseases, such as cancers, in an unprecedented resolution and speed. Computational methods to analyze and interpret large-scale biological data have become an integral part of medical research to gain knowledge that leads to personalized disease prevention, prognosis and treatment.

Particularly in cancers, genome-scale studies have revealed large molecular heterogeneity between patients and even different samples from the very same tumor [1]. Although protein expression markers have been used many years in clinics, for example in breast cancer, to classify tumors into main subtypes to guide selection of first line drug treatment, genome wide data have significantly facilitated more detailed subtyping and and identification of associated pathways and subsequently novel drug targets. In breast cancer, luminal type is characterized by high expression of estrogen receptor (ER) and/or progesterone receptor (PR), basal type by low expression of ER, PR and human epidermal growth factor receptor 2 (HER2), and high expression of basal epithelial genes [2], and triple negative (TNBC) type by low expression of all three, ER, PR and HER2 [3].

The breast cancer subtypes have different standard drug treatments based on marker protein expression: HER2 breast cancers are treated with HER2 inhibitors, such as trastuzumab, whereas luminal breast cancers are treated with adjuvant endocrine therapy, such as aromatase inhibitors. TNBC has contrasting features as there is no beneficial standard therapy for majority of patients, probably reflecting the heterogeneity of this subtype [3].

To gain a more comprehensive view to fundamental molecular level processes altered in cancer and suggest effective treatment options, several network level approaches have been suggested [4-7], such as ScorePAGE [8], SPIA [9] and DEAP [10]. These methods are based on integration of pathway topology with gene expression measurement to assign a statistical significance value to predefined pathways. Pathway topology-based approaches have been reported [6,10] to perform better than generic gene set analysis tools, such as Gene Set Enrichment Analysis (GSEA) [11]. Still, there are several limitations that need to be rectified. Firstly, most of pathway analysis methods integrate gene expression information separately for each individual canonical pathway. In reality, biological pathways are interconnected and form complex networks with shared node molecules. Thus, studying isolated pathways may lead to significantly biased results and loss of information [12]. Secondly, it is possible that only a part of a pathway is contributing to cancer progression and thus the influence of such subnetwork is challenging to identify using whole-pathway focused algorithms.

To address these two challenges, we present a novel approach called Differentially Expressed Regulation Analysis (DERA). DERA elevates the analysis of expression data to a network level instead of focusing on single genes. DERA integrates expression data with biological network instead of individual canonical pathways and identifies subnetworks that are similar active for a group of samples. These advantages of DERA are particularly useful to identify subnetworks across interconnected pathways. DERA is suitable to analyze data from small or medium size cohorts, which are challenging to analyze with statistical methods. To show the utility of our approach we applied DERA to TNBC [13,14] and high-grade serous ovarian cancer (HGS-OvCa) [15] datasets. We also compared DERA with GSEA and SPIA, which are commonly used pathway analysis methods. Our results show that DERA is able to identify biological insights specific for TNBC and HGS-OvCa. DERA shows better reproducibility and higher sensitivity in a small sample set compared with GSEA and SPIA.

## Methods

**Figure 1:**
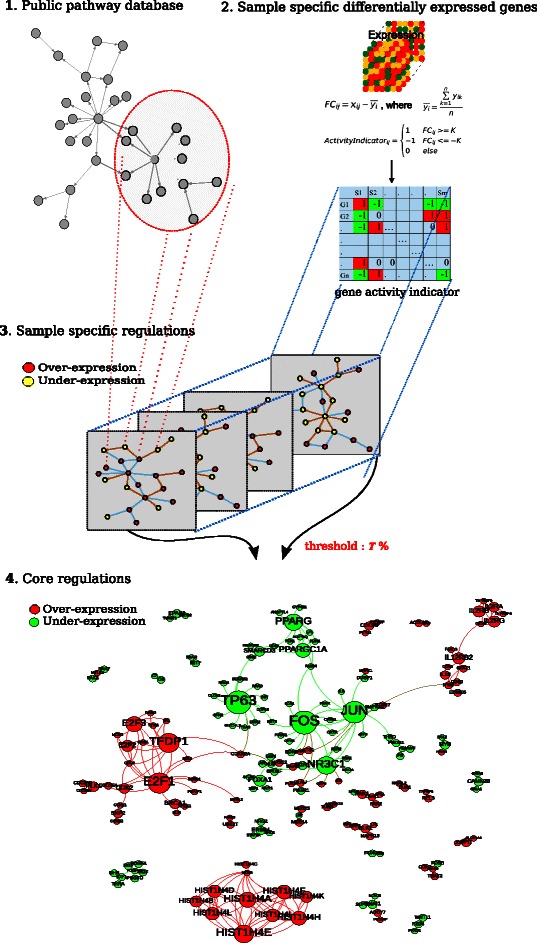
Schematic workflow of DERA. Briefly, the main steps in DERA: **1)** Extraction of the prior biological network from public database. **2)** Analysis of transcriptomics data separately for each sample to build a gene activity indicator matrix. *x*_*ij*_ and *y*_*ik*_ represent expression of gene *i* in tumor *j* and reference sample *k*. The value *n* is the number of reference samples. *K* is the threshold for the fold change. **3)** Overcoming the cross-talk issue between the pathways by using regulatory connections instead of restricting connections within an individual canonical pathway, and **4)** Identification core regulations for a group of samples, which are shared and identical at least in *T*% of samples. Node size is determined by the number of connections.

A schematic illustration of the DERA approach is shown in Figure 1. Briefly, by overlaying the expression data with the biological networks that are extracted from public databases, DERA generates sample-specific regulation networks for each individual patient, which further serve to identify the core regulations associated with phenotypes (e.g., cancer). DERA requires two types of input data: expression data (e.g., gene or protein expression data) and phenotypic information (e.g., group or subtype information). While DERA is able to integrate gene regulation or protein-protein-interaction networks with proper high-throughput molecular measurements, in our case study, we focus here on integrating gene expression data with gene regulation network.

### Public databases

To construct sample-specific networks we take advantage of publicly available databases: Pathway Commons [16], WikiPathways [17] and PINA [18]. To systematically use these sources (step 1 Figure 1), we use Moksiskaan database, which allows making networks based on gene lists [19]. Moksiskaan provides many useful application programming interfaces (APIs) to integrate information of connectivity between genes, proteins, pathways, drugs and other biological entities in the Anduril framework [20]. Pairwise connections between biological entities, instead of canonical pathways, can be exported from Moksiskaan. This allows accommodation of cross-talk between the canonical pathways and identification of even small regulations crossing the different pathways.

### Sample-specific differentially expressed genes

We analyzed level 1 Agilent two-color gene-expression microarray data for 522 primary breast tumors and 59 controls from The Cancer Genome Atlas (TCGA) repository [13] as discovery dataset (TCGA_Array), and Affymetrix Human Genome U133 Plus 2.0 Array for 43 primary breast tumors and 7 controls from GEO [21] (*accession number GSE7904* [14]) for the validation. For the further validation, we analyzed level 3 RNA-Seq data (TCGA_Seq) for 459 primary breast tumors and 55 controls from TCGA which did not overlap with microarray dataset.

We also analyzed level 1 Agilent two-color gene-expression microarray data for 572 primary ovarian cancers and 8 controls from TCGA. Out of 572 primary ovarian cancer, we selected 448 high-grade serous ovarian cancer (HGS-OvCa) according to Federation of Gynecology and Obstetrics standards.

For TCGA Agilent array breast and ovarian cancer data, expression intensities for tumors and controls were log2 transformed. This was followed by mean-centering across genes. We removed probes that a) mapped to multiple genes or b) did not map to any gene before identifying differentially expressed genes. For GEO data, gene level normalization was performed by using Robust Multi-array Average (RMA) [22].

Differentially expressed genes for each sample are identified as follows (step 2 Figure 1). Gene expression data are used to compute the gene-activity indicator matrix in which each element can take one of three values corresponding to over-expression (indicated as “1”), unchanged expression (“0”) or under-expression (“-1”) relative to control level. The relative expression (also called fold change) of a gene in a particular tumor is computed by subtracting the expression of the gene in the tumor from the mean expression of the same gene in the reference sample set. A user-defined cutoff for fold-change serves to determine the value of the gene-activity indicators, and here we adopted a frequently used two-fold difference. Sample-specific differentially expressed genes in a particular tumor patient are defined by the genes that are over-expressed or under-expressed. Sample-specific differentially expressed genes serve to induce the sample-specific regulation networks for individuals as described in the next step (step 3 Figure 1).

### Sample-specific regulations

The key concept of DERA is the generation of sample-specific regulation networks reflecting the uniqueness of individual samples at the network level. DERA is designed to improve the interpretation power of heterogeneous samples compared to many commonly used approaches. Sample-specific regulation networks are generated by overlaying sample-specific differentially expressed genes of individual samples and their gene-activity status on top of the known biological network (step 3 Figure 1). Only the regulations are selected as sample-specific regulations only if their associated genes are differentially expressed and patterns are consistent with their gene-activity status of the individual sample. For example, given a regulation where gene *A* activates the expression of gene *B*, the regulation is defined as a sample-specific regulation for the particular sample and is included in the sample-specific regulation network if both genes *A* and *B* are over-expressed or under-expressed in the particular sample. If the regulation is a gene inhibition, gene *A* and *B* are expected to have opposite expression patterns.

### Identification of core regulations

A core regulation is defined as a regulation network which is identical within a subgroup of samples and represents at least *T*% of the total number of samples (step 4 Figure 1). The empirical study of the influence of *T* in TCGA_Array and GEO cohorts illustrates that the number of regulations decreases dramatically with increasing *T* (Additional file 1: Figure S1) as expected. We used *T* value of 50, i.e., a differentially expressed regulation was required to be found and to be identical in at least 50% of the sample-specific regulation networks in order to be classified as a core regulation. In the validation, we adopted a slightly low *T* value of 40% because of a small in GEO cohort (*n*=17) and heterogeneous samples in TNBC [3]. We used the same parameter setting for the application of HGS-OvCa.

## Results and discussion

We have applied DERA into breast cancer and ovarian cancer data sets. In the breast cancer study, our aim was to identify regulations that were unique to TNBC in comparison to other breast cancer subtypes. The aims of the ovarian cancer case study were to test robustness of DERA and compare regulations identified in ovarian cancer to TNBC as they are recently suggested to share similar molecular characteristics [13].

### A case study: Triple negative breast cancer characterization with DERA

DERA was applied to breast cancer gene expression data to characterize gene regulations that occurred uniquely in TNBC in comparison to other breast cancer subtypes. We analyzed gene expression and clinical data from 366 treatment-naive breast cancer tumors from TCGA_Array data that had ER, PR and HER2 status available. From these samples, 55 samples were categorized as TNBCs (based on immunohistochemistry of ER, PR and HER2). Additionally, we used expression data from 59 samples of normal breast tissue to identify differentially expressed genes for each individual sample.

For validation of the results emerging from discovery cohort (TCGA_Array) we used data from two publications. First, we used data (GEO cohort) from David M. Livingston and colleagues who published gene expression cohort for 17 TNBC, 26 non-TNBC and seven normal breast tissue samples [14]. Second, we used RNA-seq data (TCGA_Seq cohort) from TCGA, which included 56 TNBCs and 55 normal breast tissue samples that were not present in the TCGA discovery cohort (TCGA_Array).

#### Characterization of TNBC

**Figure 2:**
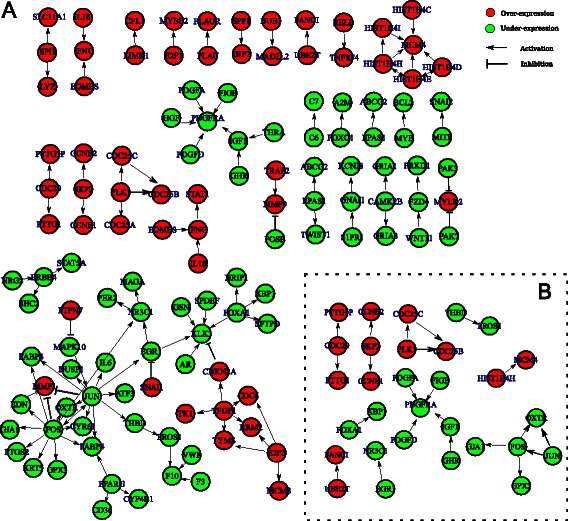
Core set of regulations and genes for TNBC.**A)** 110 core regulations that were validated in one or both of the validation cohorts. **B)** 22 regulations that were validated in both validation cohorts. Red and green represent over-expression and under-expression, respectively.

DERA identified 256 core regulations that occurred in at least half of the TNBC samples in the discovery data (TCGA_Array). Reproducibility of the results was tested in two independent cohorts resulting in verification of 110 core regulations (that consisted of 119 genes, Figure 2A) out of 254 regulations, which were validated in one or both of the validation cohorts. Out of 110 regulations, 58 regulations were validated in the GEO cohort, 74 regulations were validated in the TCGA_Seq cohort, and 22 regulations were validated in both validation cohorts (Figure 2B, Additional file 1: Figure S2).

We then used DAVID [23] to identify statistically significantly enriched pathways for the 22 regulations validated in both cohorts. Eight pathways were significantly enriched after multiple hypotheses correction (q <0.05 [24], Table 1). Cell cycle (p = 2.42×10^−6^) was the most significantly enriched pathway. This is consistent with high proliferative nature of TNBC and with previous findings [25,26]. Together, these results demonstrate that our method is able to significantly improve identification of relevant pathways and genes by combining data from multiple cohorts.

**Table 1:** **Functional enrichment analysis of 22 core regulations of TNBC validated in both cohorts**

Pathways	Count	p-value	Benjamini
Cell cycle	9	5.64×10^−8^	2.42×10^−6^
Oocyte meiosis	7	8.76×10^−6^	1.88×10^−4^
Prostate cancer	6	4.80×10^−5^	6.87×10^−4^
Pathways in cancer	9	8.12×10^−5^	8.72×10^−4^
Focal adhesion	6	2.11×10^−3^	1.80×10^−2^
Melanoma	4	4.28×10^−3^	3.03×10^−2^
Progesterone-mediated			
oocyte maturation	4	7.31×10^−3^	4.41×10^−2^
Gap junction	4	8.04×10^−3^	4.25×10^−2^

Kyoto Encyclopedia of Genes and Genomes (KEGG) pathway enrichment analysis was performed for regulations which were validated in both GEO and TCGA_Seq (adjusted p < 0.05).

The pathway analysis based on 110 core regulations indicates that the pathways are not independent but are connected at several levels. For instance, *FOS* is present in four different pathways (Myometrial Relaxation and Contraction Pathways, Oxidative Stress, Corticotropin-releasing hormone, TGF- *β* Signaling Pathway) (Additional file 1: Figure S3). Thus, by focusing just on individual pathways, the cross-talk effect would have been undetected.

The 110 core regulations consisted of 28 distinct subnetworks (Figure 2A). Subnetworks related to candidate therapeutic genes (*BCL2* [27], *FOXA1* [28], *ERBB4* [29] and *PGDG* [30]) were under-expressed while subnetworks related to cell cycle genes (*E2F1/3, CDC6, CDC20, CDC25A/B/C* and *CCNE2*) were over-expressed and promotes cell proliferation [31-33]. Another subnetwork containing the transcription factor *TFDP1*, which activates *CDKN2A, RRM2, CDC6, TK1* and *TYMS*, implicates oncogenic activity [34], proliferation [35], and invasive and metastatic potential of breast cancer [36]. Under-expression of *FOS, EDN1* and/or *JUN*, that regulate *MMP1*, and under-expression of *FOSB*, that regulates *MMP9*, are interesting findings because activation of *MMP1* and *MMP9* has been known to be involved in breast cancer initiation, invasion and metastasis [36,37].

**Figure 3:**
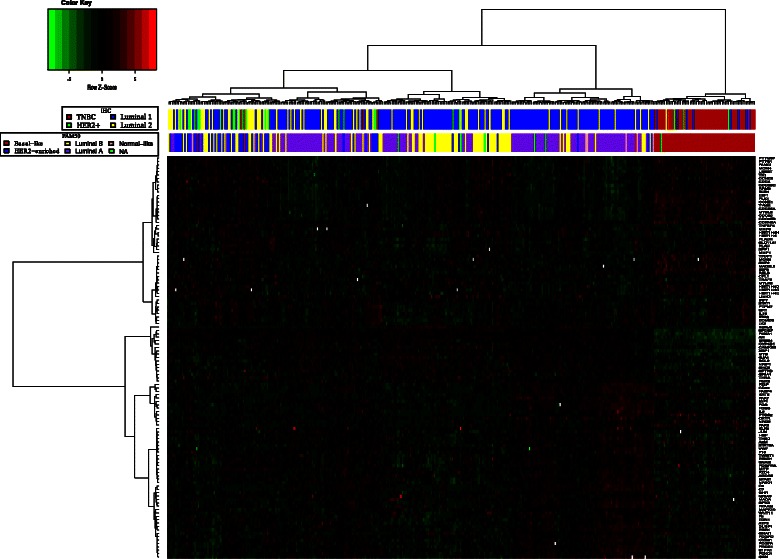
Unsupervised hierarchical clustering of breast cancer. Heatmap shows the relative gene expression compared to the median value of normal breast tissue samples of 119 differentially expressed genes. In the IHC color bar, breast cancer samples (columns) are grouped into TNBC, Luminal 1, Luminal 2 and Non-luminal HER2+ based on immunohistochemistry (IHC). In the PAM50 color bar, breast cancer samples are grouped into Basal-like, HER2-enriched, Luminal A, Luminal B and Normal-like based on gene expression. In the heatmap plot, we used euclidean distance measurement and Ward agglomeration method, and heatmap was scaled by row.

There were 119 differentially expressed genes in TNBC that contributed to the core regulations. As these 119 genes were identified in TNBC, we hypothesized that the 119 genes might be able to distinguish the TNBC cases from the other subtypes. Hierarchical clustering and heatmap representation for the 119 differentially expressed genes in the TCGA_Array (*n*=366), TCGA_Seq (*n*=319) and GEO (*n*=43) cohorts show that these differentially expressed genes are associated with TNBC phenotype and can distinguish TNBC samples from the other subtypes (Figure 3 and Additional file 1: Figure S4). In addition to categorizing breast cancer samples into the subtypes with IHC markers, we used the PAM50 [38] subtype labels. PAM50 subtype labels indicate that these 119 genes are also associated with basal-like subtype and can distinguish basal-like samples (Figure 3). The results show that there are substantial overlaps between TNBC and basal-like breast cancer and this is consistent with previous findings [39-41].

There were genes, most notably *FOXA1, AR, XBP1, SPDEF, BCL2, CYP4B1, CAMK2B, MYB, NRIP1, SHC2* and *ERBB4*, that were uniquely down-regulated in the TNBC samples while up-regulated in the other subtypes. For instance, *FOXA1* is a key determinant of estrogen receptor function [42] and negatively correlates with tumor size, tumor grade and basal-subtype, and it is an independent predictor of breast cancer survival [43]. Loss of *FOXA1* expression shifts luminal gene expression signature to basal-like and increases migration and invasion of luminal cancer cells [44]. This was consistent with our observations that *FOXA1* was down-regulated in >85% of TNBC samples while it was up-regulated in 65-93% of the other subtype samples. Furthermore, *FOXA1* was up-regulated in only 7% of the TNBC samples while it was down-regulated in 3-17% of the other subtype samples.

#### Comparison of TNBC and the other breast cancer subtypes

The prognosis for a patient with TNBC is significantly worse than a breast cancer patient having the other breast cancer subtypes [45]. Therefore, we compared the core regulations and genes in TNBC to those in the other subtypes with the aim of identifying functional modules that may convey sensitivity to current breast cancer treatments and suggest effective therapeutic targets.

We performed identical DERA analysis for Luminal 1 (*n*=219), Luminal 2 (*n*=69) and Non-luminal HER2+ (*n*=23) breast cancer subtypes as for TNBC (*n*=55) using the TCGA_Array data. The number of differentially expressed genes (*n*=189) composing the core regulations in TNBC was much higher than that in Non-luminal HER2+ (*n*=150), Luminal 1 (*n*=109) and Luminal 2 (*n*=150), which reflects the fact that in general TNBCs are more aggressive, larger in size and higher grade than the other breast cancers [3]. Furthermore, this suggests that the molecular processes involved in TNBC progression are more complex than in other subtypes.

**Figure 4:**
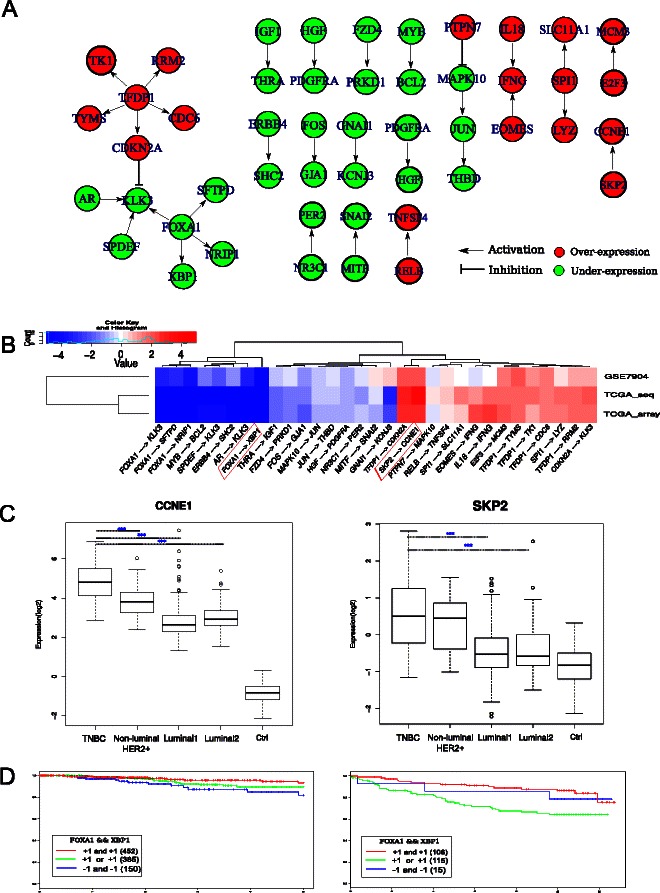
Characteristics of 31 unique regulations in TNBC identified by DERA.**A)** Differentially expressed regulations specific for TNBC. Green and red colors indicate under- and over-expression compared to median of normal breast tissues. Direction indicates gene regulation. **B)** Expression of TNBC specific regulations in terms of the signed fold-changes for the 31 regulations. Expression of a regulation is represented sum of two genes. **C)** Boxplot of log2 gene expression values of *CCNE1* and *SKP2*. TCGA_Array dataset was used to compare expression of *CCNE1* and *SKP2* in the different breast cancer subtypes. Grouping into subtypes, including TNBC (*n*=55), Luminal 1 (*n*=219), Luminal 2 (*n*=69) and Non-luminal HER2+ (*n*=23) is based on immunohistochemistry (IHC) staining. Two sided t-test was used and significance is noted by *** (*P*<1.0×10^−10^). **D)** Kaplan-Meier survival plot of *FOXA1-XBP1* regulation. Comparing patients with over-expression, neutral expression and under-expression of *FOXA1-XBP1* regulation in the TCGA (left) and GSE3494 (right) datasets. Vertical ticks represent censoring events. The *X* and *Y* axes represent follow-up time in days and the percentage of survival, respectively. The associated log-rank p-value is 0.02 in TCGA and 2.15×10^−3^ in GSE3494.

There were 256 core regulations in TNBC compared to 122 in Luminal 1, 185 in Luminal 2 and 180 in non-luminal HER2+, which may at least partly affect the poor response of TNBC to current therapeutic regimens. We identified 31 TNBC specific regulations consisting of 47 differentially expressed genes (Figure 4A), which were validated at least in one of TCGA_Seq and GEO cohorts. Five of these genes were regulated by the transcription factor *TFDP1* in TNBC. *TFDP1* related regulations were unique in TNBC. *TFDP1* is frequently amplified and associated with tumor proliferation and cell cycle progression in breast cancer [46]. Additionally, strong association between the high expression of *TFDP1* and decreased overall survival has been observed [47]. Consequently, our results suggest that the activation of *CDKN2A, RRM2, CDC6, TK1* and *TYMS* by TFDP1 might be one of the possible reasons for the aggressiveness of TNBC [34-36].

Many of 31 regulations identified with DERA were visible in independent cohorts as shown in Figure 4B. We noticed an up-regulation of *CCNE1* by *SKP2*, which is oncogenic in breast cancer [48]. High expression of *CCNE1* is independently associated with a short metastasis-free survival and the worst prognosis has been found for ER negative tumors which express high *CCNE1* [49]. A recent study has showed that inhibition of *SKP2* in prostate and lung cancer cells results in significant reduction of cancer cell proliferation and survival [50]. Our results show that although *CCNE1* is up-regulated in all the subtypes (Figure 4C), the expression difference is even higher in TNBC than in the other subtypes in all TCGA_array (Figure 4C), TCGA_Seq and GEO cohorts (Additional file 1: Figure S5). Similarly, *SKP2* had higher expression in TNBC and non-luminal HER2+ subtypes compared to the other subtypes in all the cohorts (Figure 4C, Additional file 1: Figure S5). Thus, our results suggest that higher proliferation and worse survival in TNBC might be due to up-regulation of *CCNE1* accelerated by further activation of its regulator *SKP2*. Thus, inhibition of *SKP2* should reduce cancer cell proliferation and survival in TNBC and constitute a promising target for therapeutic efforts in TNBC. Another DERA identified connection was the regulation of *XBP1* by *FOXA1*, which were significantly under-expressed as compared to non-TNBC subtypes (p-value = 3.2×10^−16^) and highly correlated (Pearson *r* =0.83, two sided p-value =2.2×10^−16^). Importantly, regulation pattern of *XBP1* by *FOXA1* was associated with breast cancer survival (log-rank p-value = 0.02) and visible in another cohort (GSE3494) (log-rank p-value = 2.15×10^−3^) (Figure 4D).

### High-grade serous ovarian cancer characterization with DERA

Ovarian cancer is the fifth leading cause of female cancer deaths in Europe [51] and more than half of the patients with high-grade serous ovarian cancer (HGS-OvCa), the most common ovarian cancer subtype, die within five years after diagnosis. It has been suggested recently that HGS-OvCa is molecularly similar to TNBC [13]. Thus, we applied DERA to expression data from 448 HGS-OvCa patients available in TCGA [15] to see whether the similarities can be seen at the network level.

Expression data from 448 HGS-OvCa samples were randomly divided into discovery set (*n* = 202) and validation set (*n* = 246), and identical DERA analysis with the TNBC analysis, i.e., cut-off *T* was 0.5 for the discovery set and 0.4 for the validation set (detailed description in Methods), was performed to the HGS-OvCa data.

The DERA analysis identified 95 differentially expressed regulations that were composed of 101 genes (Additional file 1: Figure S6). All of these 95 differentially expressed regulations were validated in the validation set (Additional file 1: Figure S7). Even using stringent threshold 0.5 for validation set (default cut-off *T* was 0.4, i.e., a differentially expressed regulation was required to be found and to be identical in at least 40% of the samples), out of 95, 87 differentially expressed regulations were validated (Additional file 1: Figure S8). This result demonstrates that the reproducibility of our method is very high when the data are measured with the same platform and sample size is relatively large.

Similarity between HGS-OvCa and TNBC has been seen at molecular level [13]. Therefore, we asked whether TNBC and HGS-OvCa share regulations. Interestingly, our results corroborate similarity between HGS-OvCa and TNBC also at the gene regulatory network level. Of the 95 differentially expressed regulations DERA identified in HGS-OvCa four regulations consisting of eight genes were also present in the set of 22 regulations (consisting of 30 genes) found to be unique in TNBC by DERA. Additionally, five genes that were consistently differentially expressed in both TNBC and HGS-OvCa, but their regulations were not validated in either TNBC or HGS-OvCa (*FOXA1, CDC25C, CCNE1, CCNE2, MCM4*). We found that cell cycle related regulation and genes (*PTTG1-CDC20, CCNE1, CCNE2, CDC25C*) were up-regulated and *PDGFRA* regulation was down-regulated in both TNBC and HGS-OvCa.

DERA identified a large subnetwork component where transcription factor *FOXM1* activates proliferation related genes (*AURKB, CCNB1/2, CENPA/F*, and *BIRC5*), and DNA repair gene *BRCA2*, was up-regulated in the HGS-OvCa. It has been reported that *FOXM1* correlates with poor patient survival and paclitaxel resistance in ovarian cancer [52]. This result indicates that DERA is able to identify reliable and potentially medically important regulations and is comparable with other methods.

### Comparison of DERA with GSEA and SPIA

We compared DERA to two existing pathway analysis methods, GSEA and SPIA. To compare sensitivity in small sample set, we used a larger dataset TCGA_Array (*n*=55) and a small cohort GEO (*n*=17). In the comparison with GSEA, we created customized gene sets using pathways from WikiPathways to identify the enriched pathways. GSEA was applied to both TCGA_Array and GEO cohorts. There were no pathways which were significantly enriched in both cohorts at false discovery rate (FDR) < 5% (Additional file 1: Table S1). Our results suggest that the performance of GSEA is highly dependent on the sample size. GSEA resulted in 10 significantly enriched pathways at FDR < 5% in the TCGA_Array cohort (Additional file 1: Table S1). However, there were no pathways identified in the GEO cohort most likely because of small sample size (Additional file 1: Table S1).

In the comparison with SPIA, four pathways were identified at FDR < 5% in both TCGA and GEO data (Cell cycle, Pathways in cancer, Focal adhesion, Melanoma) (Additional file 1: Table S2). Two pathways, Cell cycle and Focal Adhesion, were overlapped with the DERA results. However, several pathways that gave rise to identifying TNBC specific regulations were not identified by SPIA and GSEA.

## Conclusion

We have presented a novel sample-specific network analysis approach DERA and shown its utility in identifying regulations that may be behind aggressiveness and drug resistance of the TNBC and HGS-OvCa subtypes, which is rarely curable with the common anti-cancer regimens. In addition to gene expression data, DERA is applicable to proteomics data. The input for DERA is sample-specific quantitative data and phenotype information to group samples.

The application of DERA to TNBC expression data shows that it is able to identify important regulations that are related to breast cancer survival predictors and are promising therapeutic targets. One of the most promising observation is the regulation of *CCNE1* by *SKP2*. Inhibition of *SKP2* in the lung and prostate cancer cells has been shown to significantly reduce cancer cell proliferation and cancer cell survival [50]. Our result show that *SKP2* is frequently over-expressed in the TNBC and non-luminal HER2+ subtypes. Thus, based on the DERA analysis it is suggested that inhibition of *SKP2* may improve the survival of patients with TNBC and non-luminal HER2+ subtypes but probably not with luminal subtypes. Another regulation identified by DERA is connection between *XBP1* and *FOXA1*, and over-expression of both *XBP1* and *FOXA1* is significantly associated with better survival. The application of DERA to HGS-OvCa expression data corroborate the earlier finding that HGS-OvCa shares similar characteristics to TNBC at the molecular level, and our results show that the similarity is visible also at the network level. Application of DERA to TNBC and HGS-OvCa data shows that our method is able to identify reliable and potentially medically important regulations, and has high reproducibility. In the comparison with SPIA and GSEA, DERA shows better reproducibility and tolerance to small sample size.

Taken together, we have integrated high-throughput biological data to pathway information and used graph mining [53] to identify core regulations specific to phenotype. Our results with breast cancer and ovarian cancer data illustrate that DERA is capable of producing results that give a solid basis for suggesting experimentally testable hypotheses.

## Ethics statement

All results in this study are based on existing data, no new experimental material was used. The results published here are in part based upon data generated by The Cancer Genome Atlas pilot project established by the NCI and NHGRI. Information about TCGA and the investigators and institutions who constitute the TCGA research network can be found at http://cancergenome.nih.gov. The TSP study accession number in the database of Genotype and Phenotype (dbGaP) for the TCGA study used here is phs000569.v1.p7.

## Additional file

Additional file 1 **Figure S1.** Influence of cutoff. **Figure S2.** Venn diagram of differentially expressed regulations in different TNBC cohorts and their overlapping regulations. **Figure S3.** Cross-talk effect of pathways. **Figure S4.** Hierarchical clustering of breast cancer in the additional cohorts. **Figure S5.** Boxplot of log2 expression values of *CCNE1* and *SKP2* in the different breast cancer groups in the different cohorts. **Figure S6.** Core set of regulations and genes for HGS-OvCa. **Figure S7.** Venn diagram of differentially expressed regulations in HGS-OvCa discovery and validation sets, and their overlapping regulations. **Figure S8.** Venn diagram of differentially expressed regulations in HGS-OvCa discovery and validation sets, and their overlapping regulations. **Table S1.** GSEA analysis result. **Table S2.** SPIA analysis result.
