# Supplementary material for: Identification of sample-specific regulations using integrative network level analysis
Source: BMC Cancer. 2015 Apr 28;15:319. doi: 10.1186/s12885-015-1265-2 (PMC4424448; doi:10.1186/s12885-015-1265-2)
Supplement: Additional file 1 — Figure S1. Influence of cutoff. Figure S2. Venn diagram of differentially expressed regulations in different TNBC cohorts and their overlapping regulations. Figure S3. Cross-talk effect of pathways. Figure S4. Hierarchical clustering of breast cancer in the additional cohorts. Figure S5. Boxplot of log2 expression values of CCNE1 and SKP2 in the different breast cancer groups in the different cohorts. Figure S6. Core set of regulations and genes for HGS-OvCa. Figure S7. Venn diagram of differentially expressed regulations in HGS-OvCa discovery and validation sets, and their overlapping regulations. Figure S8. Venn diagram of differentially expressed regulations in HGS-OvCa discovery and validation sets, and their overlapping regulations. Table S1. GSEA analysis result. Table S2. SPIA analysis result. [file 12885_2015_1265_MOESM1_ESM.pdf]

Figure S1

Influence of cutoff in TCGA_Array (upper panel) and GEO (lower panel) cohorts. X-axis is the cutoff *T* which is used to define a core regulation. Y-axis is the number of interactions identified as core regulations.

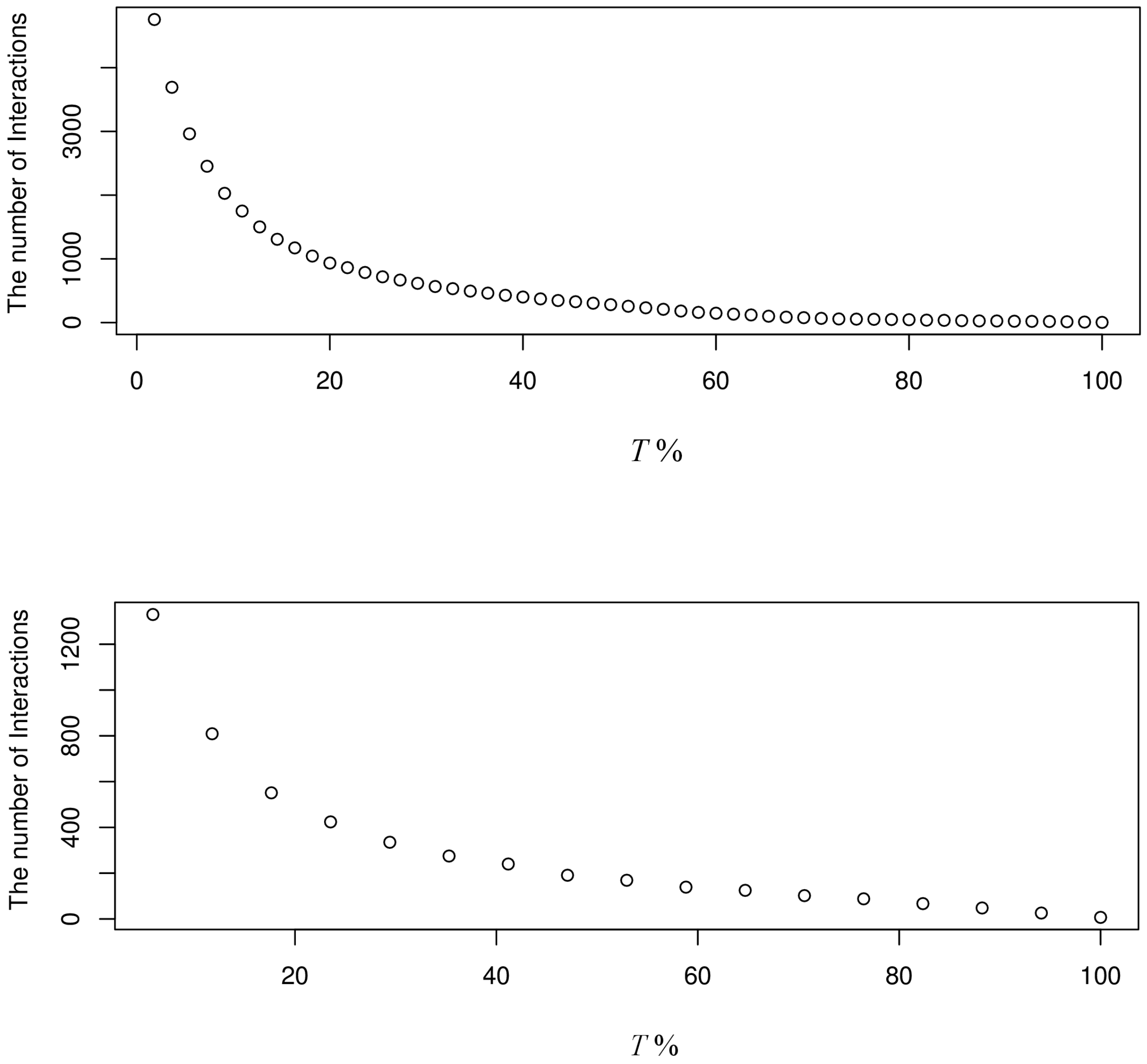

Figure S2

Venn diagram of core regulations in different TNBC cohorts and their overlapping regulations.

Venn diagram shows the overlap core regulations in TCGA_Array, GEO and TCGA_Seq cohorts.

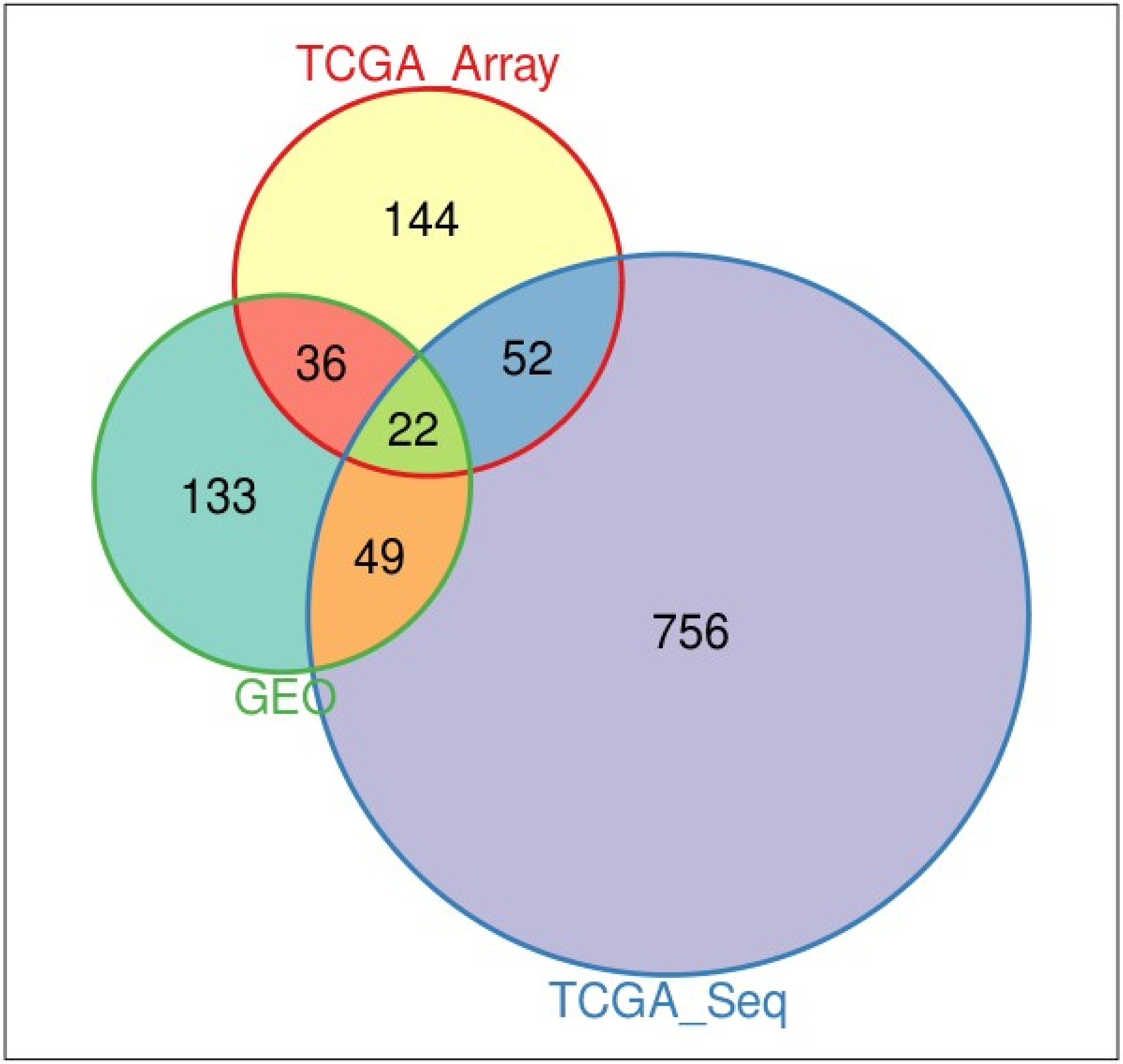

Figure S3

Cross-talk effect. Core regulations identified by DERA were mapped back to the pathways from Wikipathways database. The edge labels indicate which pathways the regulation comes from in Wikipahtways database. **WP289**="Myometrial Relaxation and Contraction Pathways", **WP408**="Oxidative Stress"; **WP560**="TGF Beta Signaling Pathway"; **WP2355**="Corticotropin-releasing hormone".

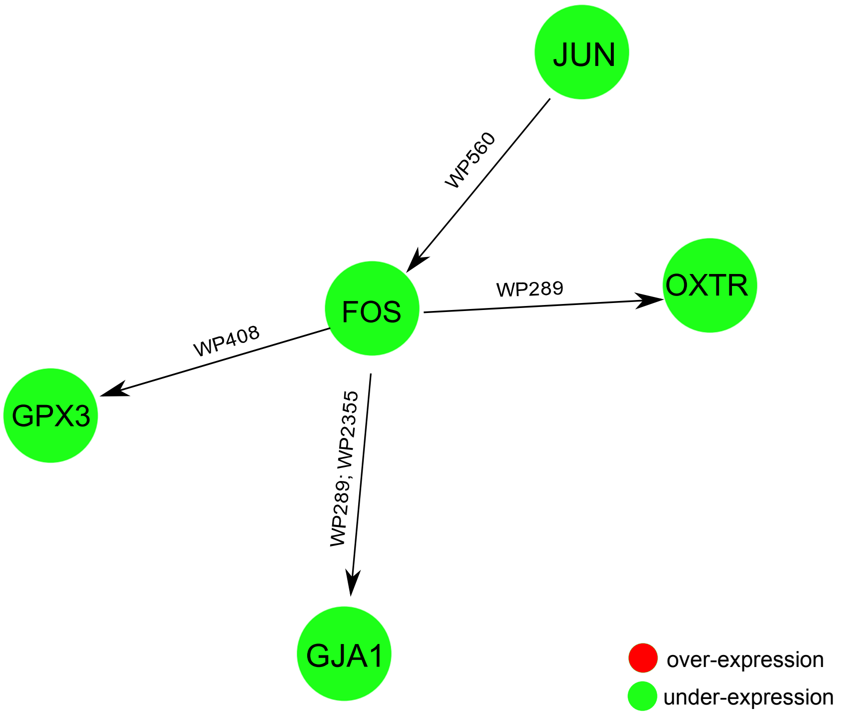

Figure S4

Hierarchical clustering of breast cancer in GEO (upper panel) and TCGA_seq (lower panel) cohorts. Heatmap shows the gene expression intensities (log2) of 119 differentially expressed genes. The heatmap was scaled by row. In the heatmap plot, we used euclidean distance measurement and ward.D agglomeration method in R.

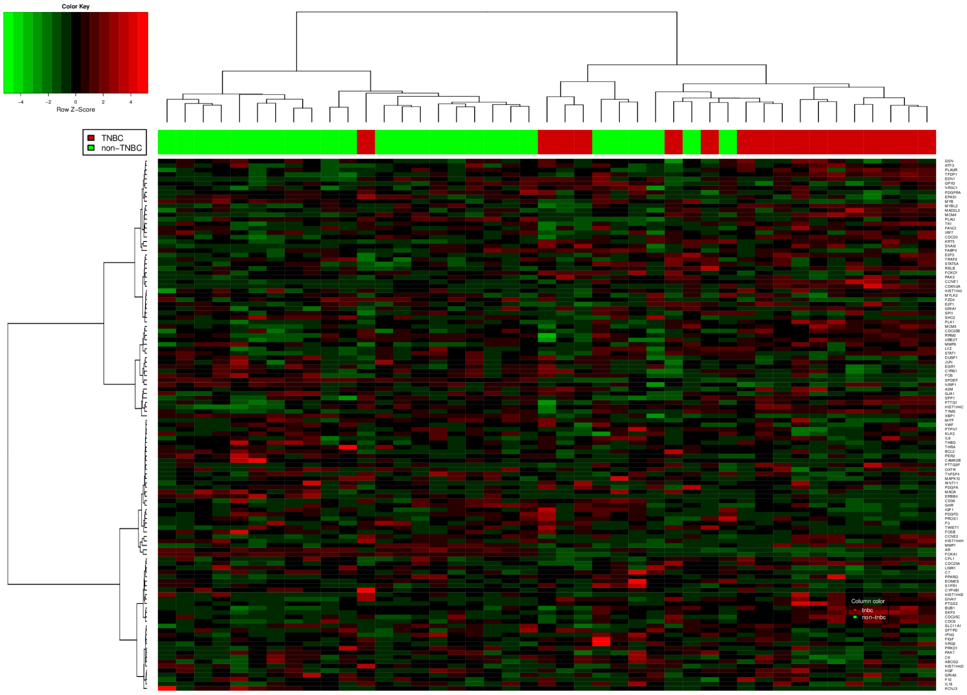

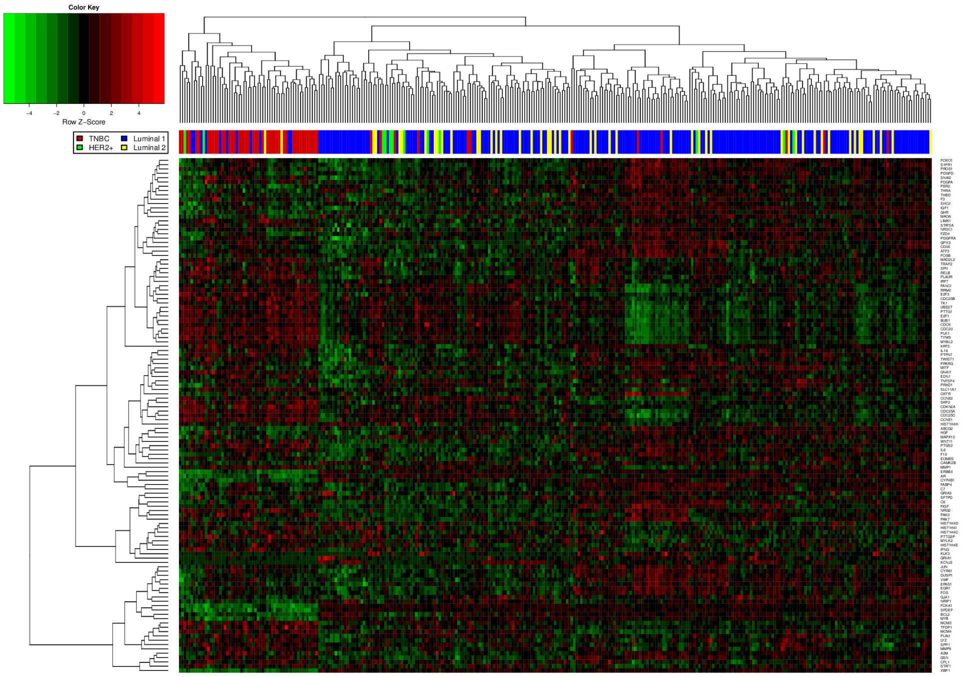

Figure S5

Boxplot of log2 expression values of *CCNE1* and *SKP2* in the different breast cancer groups in TCGA_Seq and GEO datasets. Grouping was based on immunohistochemistry (IHC) staining. Two sided t-test was used and significance was noted by *(p-value < 5.0×10^{-3} **(p-value < 5.0×10^{-6}) and *** (p-value < 5.0×10^{-10}).

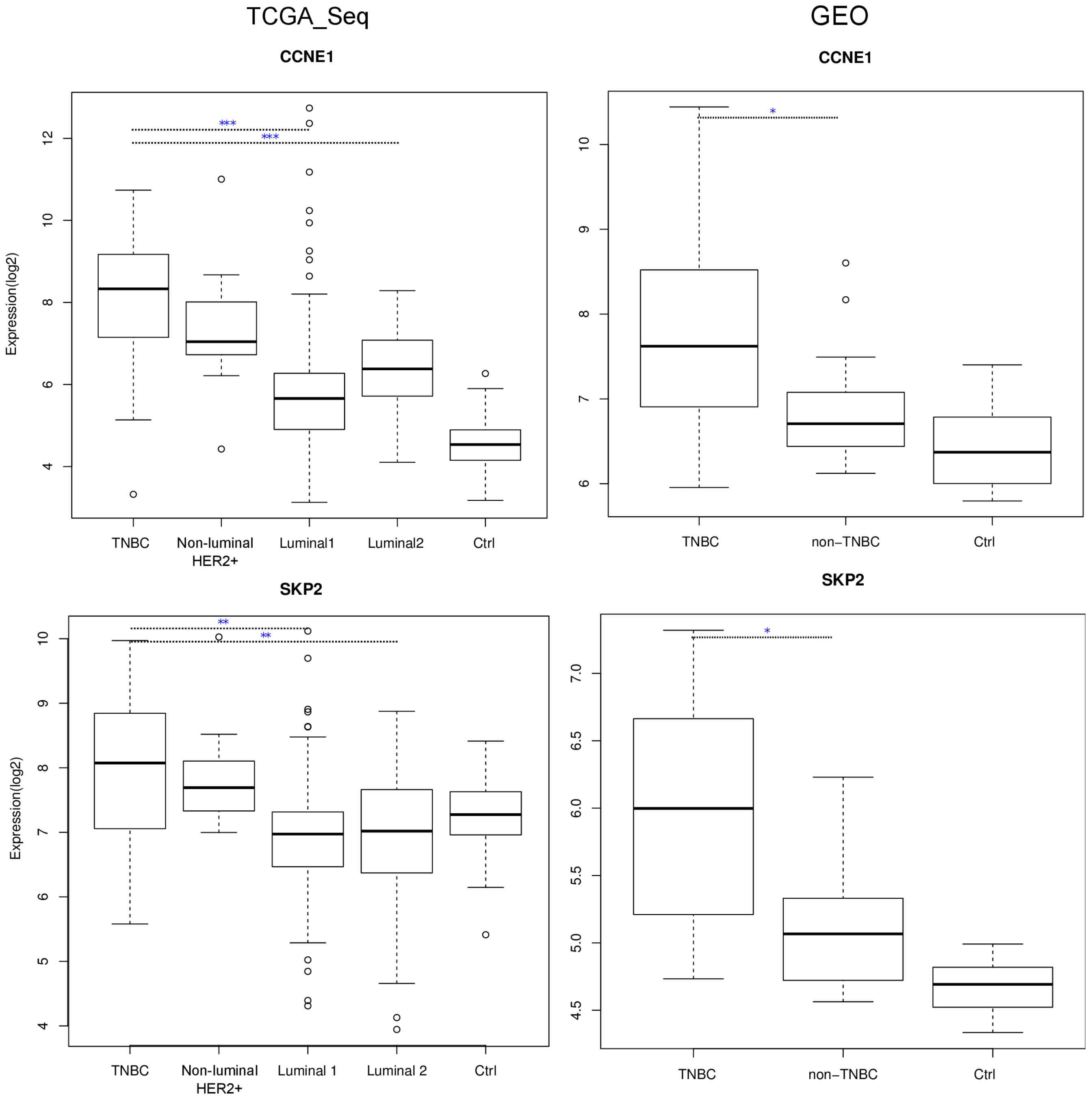

Figure S6

Core set of regulations and genes for HGS-OvCa. Red and green represent over-expression and under-expression, respectively.

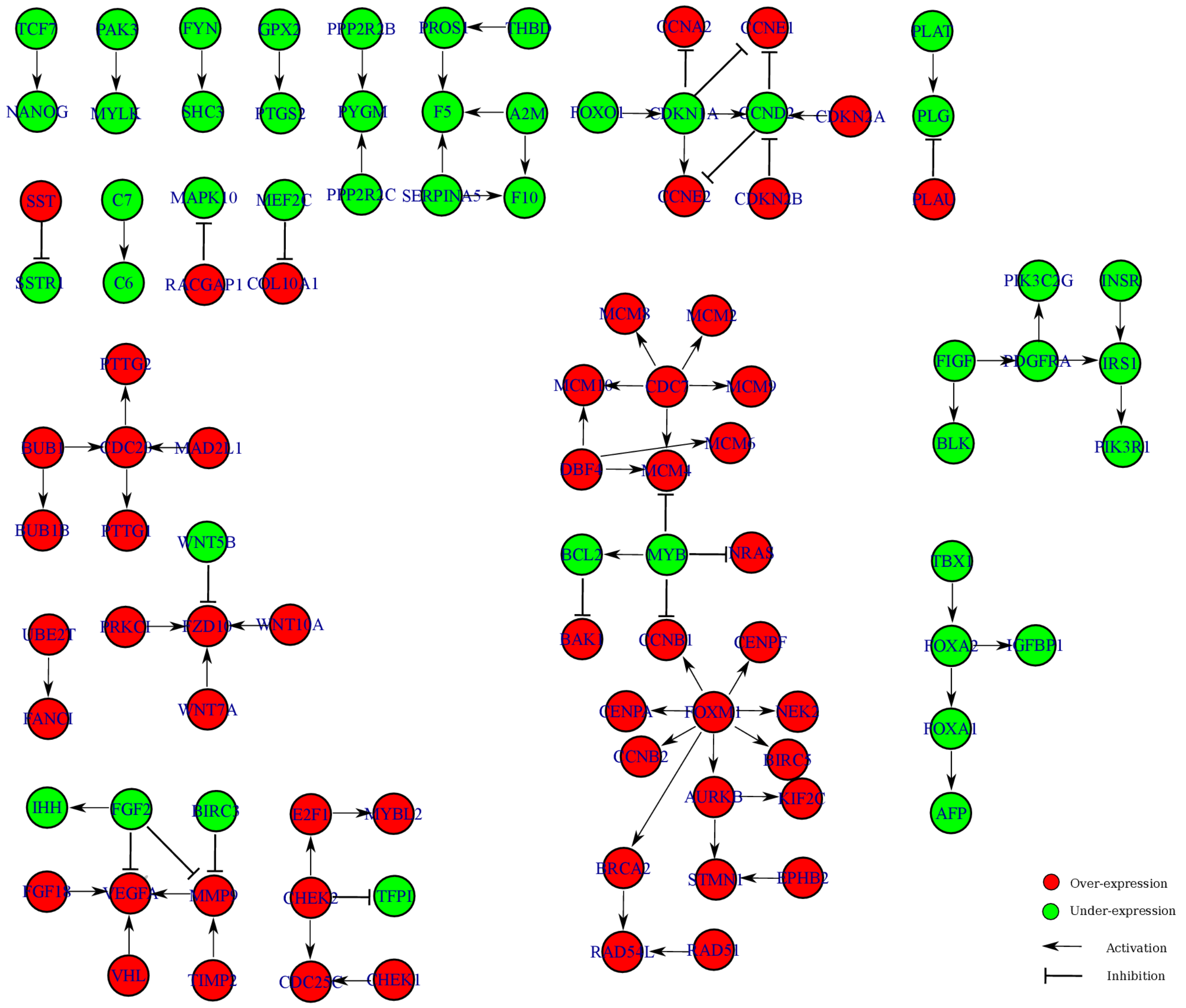

Figure S7
Venn diagram of core regulations in HGS-OvCa discovery and validation sets, and their overlapping regulations. The threshold to define core regulations was 0.4**.**

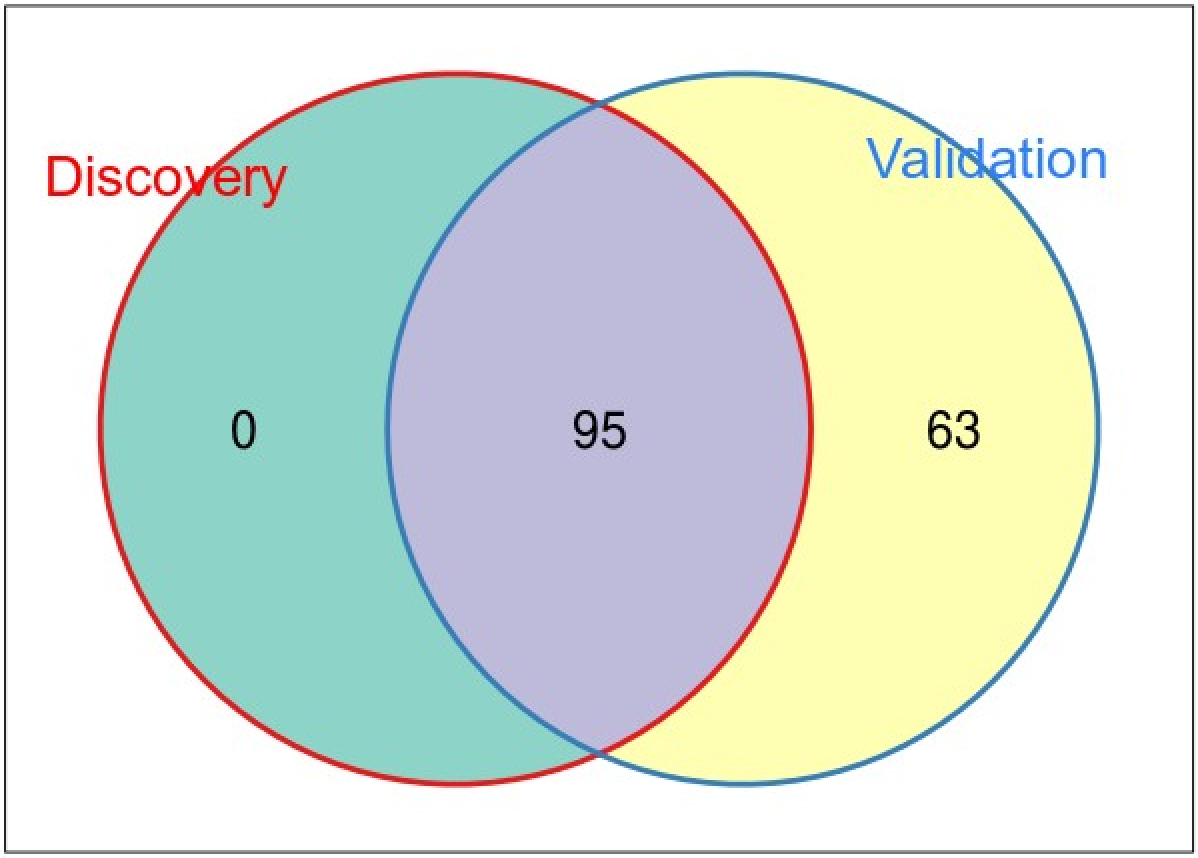

Figure S8

Venn diagram of core regulations in HGS-OvCa discovery set and validation set, and their overlapping regulations. The threshold to define core regulations was 0.5

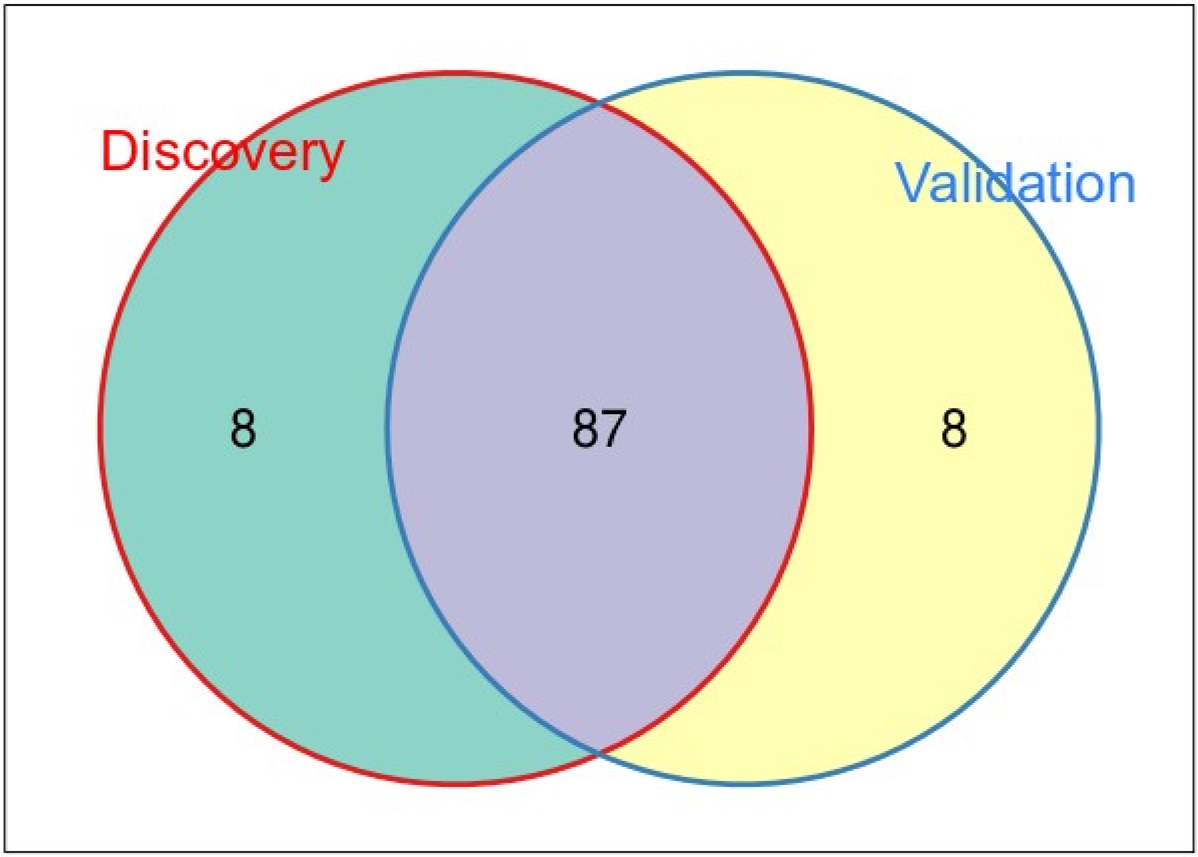

Table S1
GSEA analysis result from TCGA cohort.

ID	NAME	SIZE	ES	NES	NOM p-val	FDR q-val	FWER p-val	RANK AT MAX	LEADING EDGE
WP107	Translation Factors	29	2.49E-001	7.79E-001	7.39E-001	8.25E-001	1.00E+000	5576	tags=52%, list=38%, signal=83%
WP129	Matrix Metalloproteinases	25	4.29E-001	1.20E+000	2.51E-001	5.09E-001	9.95E-001	2953	tags=44%, list=20%, signal=55%
WP1422	Sphingolipid Metabolism	17	4.54E-001	1.31E+000	1.38E-001	4.23E-001	9.72E-001	2197	tags=29%, list=15%, signal=34%
WP1424	Globo Sphingolipid Metabolism	16	2.67E-001	7.86E-001	7.63E-001	8.34E-001	1.00E+000	4538	tags=44%, list=31%, signal=63%
WP1433	NOD pathway	34	3.55E-001	1.14E+000	2.95E-001	5.52E-001	1.00E+000	3823	tags=32%, list=26%, signal=43%
WP1449	Regulation of toll-like receptor signaling pathway	107	3.26E-001	1.11E+000	3.41E-001	5.70E-001	1.00E+000	3919	tags=37%, list=26%, signal=50%
WP1471	TOR Signaling	29	3.34E-001	9.83E-001	4.83E-001	6.53E-001	1.00E+000	4984	tags=48%, list=34%, signal=73%
WP1530	miRNA Regulation of DNA Damage Response	66	5.47E-001	1.84E+000	0.00E+000	2.46E-002	1.41E-001	2463	tags=35%, list=17%, signal=42%
WP1541	Energy Metabolism	41	2.14E-001	6.69E-001	8.74E-001	8.92E-001	1.00E+000	5275	tags=54%, list=36%, signal=83%
WP1545	miRNAs involved in DNA damage response	18	7.67E-001	2.03E+000	0.00E+000	5.51E-003	1.40E-002	1006	tags=28%, list=7%, signal=30%
WP1601	Fluoropyrimidine Activity	26	4.41E-001	1.51E+000	3.79E-002	2.31E-001	8.03E-001	1396	tags=23%, list=9%, signal=25%
WP1602	Nicotine Activity on Dopaminergic Neurons	19	2.78E-001	7.55E-001	7.95E-001	8.11E-001	1.00E+000	4292	tags=37%, list=29%, signal=52%
WP1742	TP53 Network	16	2.55E-001	7.85E-001	7.64E-001	8.26E-001	1.00E+000	2463	tags=25%, list=17%, signal=30%
WP176	Folate Metabolism	46	2.68E-001	9.96E-001	4.58E-001	6.51E-001	1.00E+000	2226	tags=24%, list=15%, signal=28%
WP1772	Apoptosis Modulation and Signaling	68	3.32E-001	1.12E+000	3.21E-001	5.69E-001	1.00E+000	4170	tags=40%, list=28%, signal=55%
WP179	Cell cycle	84	7.10E-001	2.06E+000	0.00E+000	1.08E-002	7.00E-003	739	tags=40%, list=5%, signal=42%
WP183	Proteasome Degradation	43	3.81E-001	1.21E+000	3.06E-001	5.07E-001	9.93E-001	6289	tags=74%, list=42%, signal=129%
WP195	IL-1 signaling pathway	44	2.47E-001	7.65E-001	7.30E-001	8.25E-001	1.00E+000	3768	tags=32%, list=25%, signal=43%
WP1971	Integrated Cancer pathway	32	6.50E-001	1.86E+000	0.00E+000	2.10E-002	1.06E-001	2704	tags=56%, list=18%, signal=69%
WP1984	Integrated Breast Cancer Pathway	135	4.05E-001	1.53E+000	1.21E-002	2.27E-001	7.80E-001	1553	tags=21%, list=10%, signal=24%
WP2002	miR-targeted genes in epithelium – TarBase	232	2.85E-001	1.06E+000	3.98E-001	5.91E-001	1.00E+000	3398	tags=28%, list=23%, signal=35%
WP2003	miR-targeted genes in leukocytes – TarBase	105	3.90E-001	1.32E+000	1.98E-001	4.26E-001	9.69E-001	3144	tags=36%, list=21%, signal=46%
WP2004	miR-targeted genes in lymphocytes – TarBase	354	3.23E-001	1.24E+000	1.95E-001	5.02E-001	9.89E-001	3159	tags=30%, list=21%, signal=37%
WP2005	miR-targeted genes in muscle cell – TarBase	287	2.97E-001	1.10E+000	3.36E-001	5.65E-001	1.00E+000	3159	tags=29%, list=21%, signal=36%
WP2006	miR-targeted genes in squamous cell – TarBase	107	2.85E-001	9.92E-001	4.61E-001	6.49E-001	1.00E+000	2488	tags=22%, list=17%, signal=27%
WP2012	miRs in Muscle Cell Differentiation	25	2.06E-001	6.74E-001	9.23E-001	8.96E-001	1.00E+000	3756	tags=28%, list=25%, signal=37%
WP2018	RANKL/RANK Signaling Pathway	49	2.82E-001	8.89E-001	5.97E-001	7.40E-001	1.00E+000	2797	tags=29%, list=19%, signal=35%
WP2023	Cell Differentiation – meta	18	3.93E-001	1.10E+000	3.65E-001	5.74E-001	1.00E+000	4233	tags=50%, list=29%, signal=70%
WP2032	TSH signaling pathway	58	2.96E-001	1.03E+000	4.20E-001	6.34E-001	1.00E+000	3030	tags=22%, list=20%, signal=28%
WP2035	FSH signaling pathway	23	3.14E-001	9.97E-001	4.55E-001	6.61E-001	1.00E+000	2507	tags=22%, list=17%, signal=26%
WP2036	TWEAK Signaling Pathway	40	4.71E-001	1.41E+000	1.15E-001	3.63E-001	9.17E-001	2507	tags=38%, list=17%, signal=45%
WP2037	Prolactin Signaling Pathway	65	2.92E-001	9.66E-001	5.15E-001	6.63E-001	1.00E+000	3599	tags=37%, list=24%, signal=49%
WP2038	Regulation of Microtubule Cytoskeleton	41	3.70E-001	1.21E+000	2.16E-001	5.10E-001	9.94E-001	4292	tags=49%, list=29%, signal=68%
WP205	IL-7 Signaling Pathway	22	3.18E-001	8.32E-001	6.27E-001	8.00E-001	1.00E+000	3030	tags=27%, list=20%, signal=34%
WP2100	Arylhydrocarbon receptor (AhR) signaling pathway	20	2.61E-001	8.85E-001	6.29E-001	7.36E-001	1.00E+000	2226	tags=20%, list=15%, signal=23%
WP2112	IL17 signaling pathway	27	3.31E-001	1.02E+000	4.38E-001	6.37E-001	1.00E+000	3391	tags=37%, list=23%, signal=48%
WP2203	TSLP Signaling Pathway	42	4.21E-001	1.25E+000	2.13E-001	4.84E-001	9.85E-001	3392	tags=40%, list=23%, signal=52%
WP2261	Signaling Pathways in Glioblastoma	68	2.92E-001	1.07E+000	3.50E-001	6.00E-001	1.00E+000	2704	tags=26%, list=18%, signal=32%
WP2263	Prostate cancer	97	4.02E-001	1.58E+000	9.65E-003	1.77E-001	6.83E-001	3030	tags=35%, list=20%, signal=44%
WP2272	Pathogenic Escherichia coli infection	37	4.52E-001	1.46E+000	7.71E-002	3.14E-001	8.69E-001	4058	tags=46%, list=27%, signal=63%
WP23	B Cell Receptor Signaling Pathway	80	3.27E-001	1.08E+000	3.68E-001	5.87E-001	1.00E+000	4931	tags=46%, list=33%, signal=69%
WP231	TNF alpha Signaling Pathway	72	3.32E-001	1.14E+000	3.18E-001	5.66E-001	9.99E-001	4806	tags=40%, list=32%, signal=59%
WP2324	AGE/RAGE pathway	60	3.04E-001	1.01E+000	4.64E-001	6.47E-001	1.00E+000	3816	tags=37%, list=26%, signal=49%
WP2328	Allograft Rejection	56	2.78E-001	8.53E-001	5.76E-001	7.73E-001	1.00E+000	5896	tags=52%, list=40%, signal=86%
WP2359	Parkin-Ubiquitin Proteasomal System pathway	54	4.20E-001	1.44E+000	1.13E-001	3.40E-001	8.92E-001	4086	tags=52%, list=28%, signal=71%
WP2361	Gastric cancer network 1	23	8.06E-001	1.77E+000	0.00E+000	4.14E-002	2.48E-001	1651	tags=70%, list=11%, signal=78%
WP2363	Gastric cancer network 2	23	7.40E-001	1.93E+000	0.00E+000	1.78E-002	5.70E-002	1487	tags=43%, list=10%, signal=48%
WP2369	Histone Modifications	37	4.30E-001	1.32E+000	1.58E-001	4.17E-001	9.70E-001	3122	tags=38%, list=21%, signal=48%
WP2371	Parkinsons Disease Pathway	33	4.01E-001	1.35E+000	9.59E-002	4.04E-001	9.58E-001	4170	tags=55%, list=28%, signal=76%
WP2377	Integrated Pancreatic Cancer Pathway	170	3.94E-001	1.69E+000	2.07E-003	7.38E-002	4.13E-001	2537	tags=29%, list=17%, signal=34%
WP241	One Carbon Metabolism	18	5.71E-001	1.59E+000	3.58E-002	1.78E-001	6.65E-001	1016	tags=33%, list=7%, signal=36%
WP2446	RB in Cancer	343	5.28E-001	1.92E+000	0.00E+000	1.75E-002	6.90E-002	2494	tags=37%, list=17%, signal=43%
WP2512	Integrated Lung Cancer Pathway	64	3.40E-001	1.23E+000	1.83E-001	5.07E-001	9.91E-001	2522	tags=31%, list=17%, signal=37%
WP2516	ATM Signaling Pathway	466	5.21E-001	1.83E+000	0.00E+000	2.52E-002	1.58E-001	2463	tags=36%, list=17%, signal=42%
WP2525	Trans-sulfuration and one carbon metabolism	25	4.84E-001	1.41E+000	9.92E-002	3.77E-001	9.15E-001	1181	tags=28%, list=8%, signal=30%
WP254	Apoptosis	73	3.45E-001	1.19E+000	2.48E-001	5.11E-001	9.97E-001	2507	tags=30%, list=17%, signal=36%
WP26	Signal Transduction of S1P Receptor	17	4.00E-001	1.13E+000	2.78E-001	5.58E-001	1.00E+000	2507	tags=29%, list=17%, signal=35%
WP262	EBV LMP1 signaling	19	5.26E-001	1.39E+000	1.30E-001	3.77E-001	9.31E-001	3346	tags=53%, list=23%, signal=68%
WP286	IL-3 Signaling Pathway	38	2.32E-001	7.25E-001	8.08E-001	8.44E-001	1.00E+000	3502	tags=32%, list=24%, signal=41%
WP313	Signaling of Hepatocyte Growth Factor Receptor	27	2.69E-001	7.64E-001	7.57E-001	8.17E-001	1.00E+000	1634	tags=22%, list=11%, signal=25%
WP314	FAS pathway and Stress induction of HSP regulation	34	4.61E-001	1.38E+000	1.27E-001	3.77E-001	9.37E-001	3545	tags=44%, list=24%, signal=58%
WP364	IL-6 signaling pathway	36	2.16E-001	6.46E-001	8.54E-001	9.07E-001	1.00E+000	4203	tags=36%, list=28%, signal=50%
WP382	MAPK Signaling Pathway	136	1.99E-001	7.70E-001	8.02E-001	8.27E-001	1.00E+000	1318	tags=14%, list=9%, signal=15%
WP384	Apoptosis Modulation by HSP70	18	4.27E-001	1.16E+000	2.99E-001	5.46E-001	9.98E-001	3545	tags=44%, list=24%, signal=58%
WP391	Mitochondrial Gene Expression	15	6.91E-001	1.56E+000	2.25E-002	1.89E-001	7.16E-001	3426	tags=67%, list=23%, signal=87%
WP395	IL-4 Signaling Pathway	47	2.38E-001	7.59E-001	7.88E-001	8.15E-001	1.00E+000	3030	tags=23%, list=20%, signal=29%
WP399	Wnt Signaling Pathway and Pluripotency	86	2.37E-001	9.04E-001	5.89E-001	7.23E-001	1.00E+000	4931	tags=40%, list=33%, signal=59%
WP400	p38 MAPK Signaling Pathway	28	4.10E-001	1.23E+000	2.29E-001	4.95E-001	9.91E-001	4186	tags=46%, list=28%, signal=65%
WP405	Eukaryotic Transcription Initiation	29	4.85E-001	1.37E+000	1.55E-001	3.83E-001	9.45E-001	3540	tags=45%, list=24%, signal=59%
WP411	mRNA Processing	93	4.25E-001	1.33E+000	2.28E-001	4.21E-001	9.67E-001	5355	tags=59%, list=36%, signal=92%
WP422	MAPK Cascade	19	3.33E-001	9.42E-001	5.28E-001	6.97E-001	1.00E+000	1183	tags=21%, list=8%, signal=23%
WP428	Wnt Signaling Pathway and Pluripotency	54	1.71E-001	6.21E-001	9.73E-001	9.21E-001	1.00E+000	4226	tags=28%, list=28%, signal=39%
WP437	EGF/EGFR Signaling Pathway	137	2.53E-001	9.14E-001	5.81E-001	7.36E-001	1.00E+000	3599	tags=31%, list=24%, signal=40%
WP45	G1 to S cell cycle controL	55	6.56E-001	2.03E+000	0.00E+000	7.18E-003	1.10E-002	1249	tags=36%, list=8%, signal=40%
WP453	Inflammatory Response Pathway	25	4.81E-001	1.33E+000	1.78E-001	4.34E-001	9.67E-001	4023	tags=40%, list=27%, signal=55%
WP466	DNA Replication	31	7.65E-001	1.72E+000	2.00E-003	5.87E-002	3.42E-001	2046	tags=65%, list=14%, signal=75%
WP516	Hypertrophy Model	17	2.79E-001	7.89E-001	7.27E-001	8.40E-001	1.00E+000	2799	tags=29%, list=19%, signal=36%

WP524	G13 Signaling Pathway	27	3.73E-001	1.17E+000	2.94E-001	5.43E-001	9.97E-001	4774	tags=48%, list=32%, signal=71%
WP534	Glycolysis and Gluconeogenesis	41	3.76E-001	1.30E+000	1.56E-001	4.28E-001	9.74E-001	4307	tags=49%, list=29%, signal=69%
WP581	EPO Receptor Signaling	23	3.60E-001	9.79E-001	4.85E-001	6.50E-001	1.00E+000	3502	tags=39%, list=24%, signal=51%
WP585	Interferon type I	43	2.67E-001	8.03E-001	6.86E-001	8.28E-001	1.00E+000	3599	tags=35%, list=24%, signal=46%
WP615	Senescence and Autophagy	85	2.44E-001	8.75E-001	6.18E-001	7.44E-001	1.00E+000	2209	tags=20%, list=15%, signal=23%
WP619	Type II interferon signaling	34	5.39E-001	1.29E+000	2.15E-001	4.21E-001	9.77E-001	2797	tags=41%, list=19%, signal=51%
WP623	Oxidative phosphorylation	37	1.55E-001	5.38E-001	9.02E-001	9.66E-001	1.00E+000	6539	tags=49%, list=44%, signal=87%
WP673	ErbB Signaling Pathway	46	2.47E-001	9.13E-001	6.17E-001	7.28E-001	1.00E+000	1634	tags=17%, list=11%, signal=19%
WP69	TCR Signaling Pathway	76	2.88E-001	9.10E-001	5.76E-001	7.23E-001	1.00E+000	4138	tags=38%, list=28%, signal=53%
WP707	DNA Damage Response	59	5.81E-001	1.87E+000	0.00E+000	2.33E-002	1.02E-001	2463	tags=37%, list=17%, signal=45%
WP710	DNA Damage Response (only ATM dependent)	77	2.07E-001	8.04E-001	8.20E-001	8.38E-001	1.00E+000	4226	tags=36%, list=28%, signal=51%
WP727	Monoamine Transport	28	3.50E-001	1.10E+000	3.21E-001	5.58E-001	1.00E+000	333	tags=7%, list=2%, signal=7%
WP732	Serotonin Receptor 2 and ELK-SRF/GATA4 signaling	15	3.66E-001	1.04E+000	4.02E-001	6.22E-001	1.00E+000	2404	tags=33%, list=16%, signal=40%

GSEA analysis result from GEO cohort.

ID	NAME	SIZE	ES	NES	NOM p-val	FDR q-val	FWER p-val	RANK AT MAX	LEADING EDGE
WP1530	miRNA Regulation of DNA Damage Response	71	5.59E-001	1.48E+000	1.15E-002	2.43E-001	7.99E-001	1432	tags=28%, list=8%, signal=31%
WP179	Cell cycle	95	7.42E-001	1.49E+000	9.67E-003	2.59E-001	7.87E-001	1329	tags=42%, list=8%, signal=45%
WP183	Proteasome Degradation	51	4.97E-001	1.50E+000	1.08E-001	2.74E-001	7.60E-001	4223	tags=47%, list=24%, signal=62%
WP2446	RB in Cancer	391	5.49E-001	1.53E+000	2.95E-002	2.75E-001	6.81E-001	2084	tags=31%, list=12%, signal=35%
WP404	Nucleotide Metabolism	15	7.48E-001	1.51E+000	1.74E-002	2.83E-001	7.32E-001	3408	tags=60%, list=20%, signal=75%
WP2361	Gastric cancer network 1	25	8.77E-001	1.45E+000	3.94E-003	2.93E-001	8.61E-001	1175	tags=64%, list=7%, signal=69%
WP1971	Integrated Cancer pathway	33	7.25E-001	1.54E+000	9.60E-003	3.35E-001	6.74E-001	1639	tags=45%, list=9%, signal=50%
WP1601	Fluoropyrimidine Activity	28	6.20E-001	1.40E+000	6.88E-002	3.52E-001	9.26E-001	1920	tags=36%, list=11%, signal=40%
WP466	DNA Replication	37	8.11E-001	1.40E+000	4.72E-002	3.65E-001	9.17E-001	1257	tags=51%, list=7%, signal=55%
WP2525	Trans-sulfuration and one carbon metabolism	27	5.73E-001	1.37E+000	1.26E-001	4.01E-001	9.51E-001	2386	tags=37%, list=14%, signal=43%
WP2363	Gastric cancer network 2	29	8.24E-001	1.54E+000	3.82E-003	4.14E-001	6.43E-001	1136	tags=52%, list=7%, signal=55%
WP2359	Parkin-Ubiquitin Proteasomal System pathway	61	4.04E-001	1.34E+000	1.24E-001	4.29E-001	9.65E-001	4008	tags=34%, list=23%, signal=45%
WP391	Mitochondrial Gene Expression	16	4.21E-001	1.23E+000	1.94E-001	4.37E-001	9.93E-001	3393	tags=31%, list=19%, signal=39%
WP2371	Parkinsons Disease Pathway	36	4.77E-001	1.23E+000	1.96E-001	4.42E-001	9.93E-001	1391	tags=19%, list=8%, signal=21%
WP314	FAS pathway and Stress induction of HSP regulation	36	4.18E-001	1.22E+000	2.58E-001	4.43E-001	9.93E-001	3475	tags=39%, list=20%, signal=48%
WP2038	Regulation of Microtubule Cytoskeleton	42	3.77E-001	1.23E+000	1.82E-001	4.59E-001	9.93E-001	1175	tags=12%, list=7%, signal=13%
WP2023	Cell Differentiation – meta	20	5.16E-001	1.20E+000	1.98E-001	4.61E-001	9.95E-001	2779	tags=40%, list=16%, signal=48%
WP623	Oxidative phosphorylation	49	3.05E-001	1.23E+000	2.42E-001	4.74E-001	9.93E-001	6200	tags=43%, list=36%, signal=66%
WP1449	Regulation of toll-like receptor signaling pathway	123	4.37E-001	1.24E+000	2.14E-001	4.93E-001	9.93E-001	2002	tags=21%, list=11%, signal=24%
WP2029	Cell Differentiation – Index	15	5.45E-001	1.16E+000	2.78E-001	5.09E-001	9.99E-001	2581	tags=40%, list=15%, signal=47%
WP262	EBV LMP1 signaling	19	5.70E-001	1.24E+000	2.47E-001	5.16E-001	9.93E-001	1898	tags=37%, list=11%, signal=41%
WP727	Monoamine Transport	29	4.30E-001	1.16E+000	2.20E-001	5.18E-001	9.98E-001	1402	tags=21%, list=8%, signal=22%
WP45	G1 to S cell cycle controL	63	6.91E-001	1.56E+000	3.91E-003	5.19E-001	5.95E-001	1329	tags=37%, list=8%, signal=39%
WP241	One Carbon Metabolism	23	4.42E-001	1.24E+000	2.20E-001	5.28E-001	9.92E-001	609	tags=17%, list=3%, signal=18%
WP1984	Integrated Breast Cancer Pathway	146	3.10E-001	1.14E+000	2.28E-001	5.29E-001	9.99E-001	2068	tags=18%, list=12%, signal=21%
WP619	Type II interferon signaling	35	6.80E-001	1.28E+000	2.25E-001	5.39E-001	9.89E-001	1573	tags=34%, list=9%, signal=38%
WP129	Matrix Metalloproteinases	28	5.52E-001	1.24E+000	1.68E-001	5.53E-001	9.92E-001	2017	tags=32%, list=12%, signal=36%
WP405	Eukaryotic Transcription Initiation	34	3.91E-001	1.29E+000	2.29E-001	5.56E-001	9.88E-001	4190	tags=35%, list=24%, signal=46%
WP75	Toll-like receptor signaling pathway	87	4.74E-001	1.25E+000	2.18E-001	5.67E-001	9.92E-001	2002	tags=24%, list=11%, signal=27%
WP2012	miRs in Muscle Cell Differentiation	28	4.11E-001	1.25E+000	1.53E-001	5.78E-001	9.92E-001	3114	tags=25%, list=18%, signal=30%
WP1424	Globo Sphingolipid Metabolism	18	5.01E-001	1.10E+000	3.51E-001	6.06E-001	1.00E+000	2322	tags=28%, list=13%, signal=32%
WP1433	NOD pathway	37	3.91E-001	1.07E+000	3.80E-001	6.19E-001	1.00E+000	4898	tags=41%, list=28%, signal=56%
WP2272	Pathogenic Escherichia coli infection	44	3.41E-001	1.02E+000	4.21E-001	6.23E-001	1.00E+000	4790	tags=39%, list=28%, signal=53%
WP1742	TP53 Network	17	4.79E-001	1.02E+000	4.24E-001	6.37E-001	1.00E+000	1432	tags=29%, list=8%, signal=32%
WP2377	Integrated Pancreatic Cancer Pathway	183	3.09E-001	1.07E+000	3.39E-001	6.37E-001	1.00E+000	1512	tags=19%, list=9%, signal=20%
WP2263	Prostate cancer	102	3.22E-001	1.03E+000	4.03E-001	6.45E-001	1.00E+000	1743	tags=22%, list=10%, signal=24%
WP2369	Histone Modifications	40	3.22E-001	1.05E+000	3.65E-001	6.49E-001	1.00E+000	1717	tags=15%, list=10%, signal=17%
WP2203	TSLP Signaling Pathway	45	4.06E-001	1.03E+000	4.25E-001	6.54E-001	1.00E+000	2359	tags=29%, list=14%, signal=33%
WP1772	Apoptosis Modulation and Signaling	72	3.46E-001	9.99E-001	4.30E-001	6.55E-001	1.00E+000	1710	tags=19%, list=10%, signal=21%
WP2328	Allograft Rejection	65	3.82E-001	9.81E-001	4.77E-001	6.58E-001	1.00E+000	1402	tags=15%, list=8%, signal=17%
WP2036	TWEAK Signaling Pathway	40	3.60E-001	9.88E-001	4.86E-001	6.60E-001	1.00E+000	1898	tags=23%, list=11%, signal=25%
WP384	Apoptosis Modulation by HSP70	19	3.64E-001	1.03E+000	4.16E-001	6.70E-001	1.00E+000	3347	tags=32%, list=19%, signal=39%
WP2004	miR-targeted genes in lymphocytes – TarBase	388	2.47E-001	9.67E-001	5.16E-001	6.71E-001	1.00E+000	2570	tags=19%, list=15%, signal=22%
WP2003	miR-targeted genes in leukocytes – TarBase	117	2.79E-001	9.50E-001	5.25E-001	6.89E-001	1.00E+000	2056	tags=16%, list=12%, signal=18%
WP1545	miRNAs involved in DNA damage response	19	3.90E-001	9.11E-001	5.96E-001	7.50E-001	1.00E+000	1329	tags=26%, list=8%, signal=28%
WP53	ID signaling pathway	15	3.48E-001	8.50E-001	6.72E-001	7.55E-001	1.00E+000	887	tags=13%, list=5%, signal=14%
WP422	MAPK Cascade	27	2.79E-001	8.55E-001	6.52E-001	7.62E-001	1.00E+000	1865	tags=19%, list=11%, signal=21%
WP2002	miR-targeted genes in epithelium – TarBase	255	2.26E-001	8.88E-001	7.12E-001	7.64E-001	1.00E+000	1752	tags=13%, list=10%, signal=15%
WP231	TNF alpha Signaling Pathway	78	2.70E-001	8.59E-001	5.99E-001	7.69E-001	1.00E+000	2002	tags=17%, list=11%, signal=19%
WP1602	Nicotine Activity on Dopaminergic Neurons	20	3.32E-001	8.74E-001	6.41E-001	7.73E-001	1.00E+000	1653	tags=10%, list=9%, signal=11%
WP1471	TOR Signaling	30	2.55E-001	8.89E-001	6.43E-001	7.77E-001	1.00E+000	4494	tags=40%, list=26%, signal=54%
WP400	p38 MAPK Signaling Pathway	31	2.91E-001	8.61E-001	6.45E-001	7.81E-001	1.00E+000	1512	tags=16%, list=9%, signal=18%
WP1993	Angiogenesis overview	61	2.56E-001	8.20E-001	7.50E-001	7.92E-001	1.00E+000	2188	tags=21%, list=13%, signal=24%
WP2032	TSH signaling pathway	60	2.16E-001	7.69E-001	8.31E-001	8.31E-001	1.00E+000	1743	tags=13%, list=10%, signal=15%
WP534	Glycolysis and Gluconeogenesis	45	2.25E-001	7.53E-001	8.08E-001	8.42E-001	1.00E+000	4164	tags=29%, list=24%, signal=38%
WP111	Electron Transport Chain	80	1.62E-001	7.72E-001	6.99E-001	8.42E-001	1.00E+000	6317	tags=43%, list=36%, signal=66%
WP455	GPCRs, Class A Rhodopsin-like	220	2.14E-001	7.80E-001	7.48E-001	8.44E-001	1.00E+000	5329	tags=42%, list=31%, signal=60%
WP411	mRNA Processing	114	1.88E-001	7.03E-001	7.73E-001	8.94E-001	1.00E+000	3108	tags=18%, list=18%, signal=22%
WP23	B Cell Receptor Signaling Pathway	89	1.94E-001	6.37E-001	9.20E-001	9.44E-001	1.00E+000	3115	tags=19%, list=18%, signal=23%
WP197	Cholesterol Biosynthesis	15	2.97E-001	6.04E-001	8.61E-001	9.51E-001	1.00E+000	1632	tags=20%, list=9%, signal=22%
WP707	DNA Damage Response	62	6.33E-001	1.56E+000	1.91E-003	1.00E+000	5.92E-001	1432	tags=32%, list=8%, signal=35%

Table S2

SPIA analysis result from TCGA cohort

Name	ID	pSize	NDE	pNDE	tA	pPERT	pG	pGFdr	pGFWER	Status
Fanconi anemia pathway	hsa03460	47	31	9.7357E-007	8.02336342	0.066	1.1284E-006	0.000070452	0.000154585	Activated
Cell cycle	hsa04110	115	63	0.000000125	-9.67231275	0.632	0.000001371	0.000070452	0.000187821	Inhibited
Pathways in cancer	hsa05200	306	113	0.017909194	-155.4885011	0.000005	1.5427E-006	0.000070452	0.000211356	Inhibited
Focal adhesion	hsa04510	191	69	0.081848818	-122.1651974	0.000005	6.4288E-006	0.000181735	0.000880745	Inhibited
Melanoma	hsa05218	68	27	0.084624188	-107.5915319	0.000005	6.6327E-006	0.000181735	0.000908677	Inhibited
Adipocytokine signaling pathway	hsa04920	65	21	0.469372224	-42.33552693	0.000005	3.2768E-005	0.0007482	0.004489201	Inhibited
Tuberculosis	hsa05152	151	45	0.673711462	61.9375877	0.000005	4.5816E-005	0.000896681	0.006276769	Activated
Calcium signaling pathway	hsa04020	173	49	0.815760754	-55.82882243	0.000005	5.4696E-005	0.000936662	0.007493292	Inhibited
Transcriptional misregulation in cancer	hsa05202	152	63	0.004667336	1.303571817	0.124	0.00489312	0.074484164	0.670357477	Activated
Chemokine signaling pathway	hsa04062	171	62	0.089150428	62.37557446	0.008	0.005880893	0.080568231	0.805682312	Activated
Aldosterone-regulated sodium reabsorption	hsa04960	36	12	0.452364018	-17.26399834	0.004	0.013235681	0.164844388	1	Inhibited
Viral carcinogenesis	hsa05203	171	71	0.00265599	-0.415659165	0.831	0.015706061	0.179310865	1	Inhibited
Gap junction	hsa04540	85	33	0.081823582	-43.52565232	0.03	0.01720688	0.181334043	1	Inhibited
Oocyte meiosis	hsa04114	99	42	0.011806101	15.27572958	0.357	0.027266066	0.266817934	1	Activated
Axon guidance	hsa04360	122	49	0.021903655	-14.88895624	0.288	0.038265208	0.340443611	1	Inhibited
RNA transport	hsa03013	129	54	0.006604644	0	1	0.039759838	0.340443611	1	Inhibited
Prostate cancer	hsa05215	81	30	0.154443272	-39.96134343	0.053	0.047519996	0.382955265	1	Inhibited
Neuroactive ligand-receptor interaction	hsa04080	253	68	0.942292537	-10.66186959	0.011	0.057727003	0.439366634	1	Inhibited
Systemic lupus erythematosus	hsa05322	68	30	0.016721444	0.855360777	0.836	0.073672656	0.492054136	1	Activated
Pathogenic Escherichia coli infection	hsa05130	44	14	0.521012921	24.87803024	0.027	0.074049102	0.492054136	1	Activated
Bladder cancer	hsa05219	41	18	0.058782366	10.88934623	0.246	0.075719854	0.492054136	1	Activated
Progesterone-mediated oocyte maturation	hsa04914	80	32	0.058854985	10.34021408	0.259	0.079015993	0.492054136	1	Activated
NF-kappa B signaling pathway	hsa04064	83	27	0.436785393	24.98257058	0.042	0.091695561	0.546103496	1	Activated
Malaria	hsa05144	44	19	0.062605609	-1.661635031	0.309	0.095667766	0.546103496	1	Inhibited
ECM-receptor interaction	hsa04512	78	27	0.293933222	-12.62490847	0.07	0.10048295	0.550646566	1	Inhibited
T cell receptor signaling pathway	hsa04660	102	37	0.157698062	21.97459258	0.138	0.105059285	0.553581619	1	Activated
Sulfur relay system	hsa04122	9	6	0.030653919	0.930972017	0.788	0.114091513	0.569415532	1	Activated
p53 signaling pathway	hsa04115	64	27	0.041080057	3.054484344	0.608	0.11713593	0.569415532	1	Activated
Herpes simplex infection	hsa05168	155	55	0.142346368	17.30229907	0.19	0.12468722	0.569415532	1	Activated
Protein processing in endoplasmic reticulum	hsa04141	151	55	0.097058029	7.492304594	0.288	0.127946302	0.569415532	1	Activated
RNA degradation	hsa03018	66	28	0.035258412	-0.447281468	0.814	0.130610861	0.569415532	1	Inhibited
HTLV-I infection	hsa05166	236	86	0.047744562	-8.706942077	0.639	0.136976496	0.569415532	1	Inhibited
Intestinal immune network for IgA production	hsa04672	37	11	0.636686203	4.179740271	0.048	0.137158486	0.569415532	1	Activated
Salivary secretion	hsa04970	79	27	0.321406548	-10.81355745	0.106	0.149200986	0.599131551	1	Inhibited
mTOR signaling pathway	hsa04150	57	22	0.143744993	-5.116816933	0.245	0.153062805	0.599131551	1	Inhibited
Tight junction	hsa04530	124	44	0.173795917	10.60802871	0.22	0.163034523	0.620436936	1	Activated
Endocrine and other factor-regulated calcium reabsorption	hsa04961	46	13	0.717422396	-12.80777329	0.056	0.169320057	0.626941832	1	Inhibited
PPAR signaling pathway	hsa03320	66	26	0.09713667	-1.882460209	0.436	0.176256694	0.635451767	1	Inhibited
Complement and coagulation cascades	hsa04610	64	26	0.069501459	-9.022550245	0.701	0.19593713	0.68829197	1	Inhibited
VEGF signaling pathway	hsa04370	68	20	0.668284074	18.57476033	0.082	0.213940717	0.717562696	1	Activated
Alcoholism	hsa05034	119	42	0.191430917	20.4060478	0.293	0.217671809	0.717562696	1	Activated
Circadian rhythm - mammal	hsa04710	21	9	0.177791954	6.052166638	0.32	0.219982724	0.717562696	1	Activated
Cytokine-cytokine receptor interaction	hsa04060	233	79	0.202930934	-10.87534073	0.31	0.236918313	0.73479369	1	Inhibited
Dilated cardiomyopathy	hsa05414	85	32	0.121866238	1.804225782	0.524	0.239537072	0.73479369	1	Activated
Small cell lung cancer	hsa05222	78	26	0.382034884	-20.62495605	0.175	0.247716085	0.73479369	1	Inhibited
African trypanosomiasis	hsa05143	31	10	0.516418832	3.474982488	0.135	0.25539384	0.73479369	1	Activated
Fc gamma R-mediated phagocytosis	hsa04666	83	29	0.264827785	15.93600282	0.264	0.255920877	0.73479369	1	Activated
Gastric acid secretion	hsa04971	71	21	0.658772486	8.219646297	0.107	0.257445964	0.73479369	1	Activated
Thyroid cancer	hsa05216	28	9	0.527689698	8.017298506	0.142	0.269093705	0.752364033	1	Activated
Basal cell carcinoma	hsa05217	53	13	0.886362089	-23.54654927	0.09	0.281483587	0.771265029	1	Inhibited
Pertussis	hsa05133	66	20	0.607877022	14.24660395	0.14	0.294787151	0.789009119	1	Activated
Rheumatoid arthritis	hsa05323	76	27	0.241708998	2.405086763	0.36	0.299477914	0.789009119	1	Activated
Glioma	hsa05214	61	20	0.441340979	-22.12269579	0.207	0.309973902	0.793254385	1	Inhibited
Mineral absorption	hsa04978	46	19	0.094914489	0	1	0.318417125	0.793254385	1	Inhibited
Fc epsilon RI signaling pathway	hsa04664	71	18	0.885140399	21.21087278	0.108	0.320017528	0.793254385	1	Activated
Toll-like receptor signaling pathway	hsa04620	88	26	0.669287039	18.2671624	0.156	0.340314307	0.793254385	1	Activated
Leishmaniasis	hsa05140	57	18	0.524799808	9.850109279	0.199	0.340373916	0.793254385	1	Activated
Epstein-Barr virus infection	hsa05169	169	51	0.640868314	12.20983477	0.165	0.343321025	0.793254385	1	Activated
Type I diabetes mellitus	hsa04940	34	6	0.976044729	1.649466079	0.115	0.357733132	0.793254385	1	Activated
Antigen processing and presentation	hsa04612	49	11	0.934033144	4.252442334	0.123	0.36347821	0.793254385	1	Activated
Graft-versus-host disease	hsa05332	28	5	0.964254569	1.649466079	0.12	0.365259262	0.793254385	1	Activated
Cytosolic DNA-sensing pathway	hsa04623	48	15	0.55090301	3.694906033	0.211	0.366401054	0.793254385	1	Activated
Arrhythmogenic right ventricular cardiomyopathy (ARVC)	hsa05412	69	25	0.217311989	0.765470184	0.541	0.369245592	0.793254385	1	Activated
Allograft rejection	hsa05330	29	4	0.991474734	1.649466079	0.123	0.378553309	0.793254385	1	Activated
NOD-like receptor signaling pathway	hsa04621	54	20	0.215294106	5.132373598	0.575	0.382416781	0.793254385	1	Activated
B cell receptor signaling pathway	hsa04662	71	21	0.658772486	14.50325276	0.192	0.388007966	0.793254385	1	Activated
Natural killer cell mediated cytotoxicity	hsa04650	109	28	0.913160971	42.47543312	0.143	0.396414854	0.793254385	1	Activated
Renal cell carcinoma	hsa05211	63	24	0.147462912	-0.821250432	0.913	0.404600738	0.793254385	1	Inhibited
Shigellosis	hsa05131	55	15	0.778317796	10.21170913	0.174	0.406189845	0.793254385	1	Activated
Maturity onset diabetes of the young	hsa04950	21	3	0.979160591	4.508222936	0.139	0.407539752	0.793254385	1	Activated
Viral myocarditis	hsa05416	60	18	0.62679869	5.738551224	0.22	0.411102637	0.793254385	1	Activated
Autoimmune thyroid disease	hsa05320	39	6	0.992829577	1.649466079	0.147	0.42682169	0.802693638	1	Activated
Salmonella infection	hsa05132	74	19	0.876702344	9.962973348	0.167	0.427712668	0.802693638	1	Activated
Vascular smooth muscle contraction	hsa04270	106	32	0.624427775	-18.19353216	0.242	0.436672579	0.80843437	1	Inhibited
Amyotrophic lateral sclerosis (ALS)	hsa05014	48	13	0.777245749	7.708939773	0.208	0.456259505	0.825772315	1	Activated
Chronic myeloid leukemia	hsa05220	69	23	0.393885825	8.679949038	0.413	0.458092671	0.825772315	1	Activated
Lysosome	hsa04142	109	39	0.174915149	0	1	0.479871716	0.833053781	1	Inhibited
MAPK signaling pathway	hsa04010	252	84	0.249944066	-5.225822603	0.705	0.482125431	0.833053781	1	Inhibited

ErbB signaling pathway	hsa04012	83	26	0.531045325	-21.04685178	0.342	0.49143016	0.833053781	1	Inhibited
Vibrio cholerae infection	hsa05110	49	12	0.880512647	2.616111319	0.207	0.492535447	0.833053781	1	Activated
Prion diseases	hsa05020	35	13	0.276791607	2.076415149	0.734	0.526956605	0.871568658	1	Activated
Apoptosis	hsa04210	82	20	0.929507968	15.75753953	0.222	0.532008971	0.871568658	1	Activated
Pancreatic cancer	hsa05212	68	24	0.270838104	2.994655129	0.804	0.549695616	0.871568658	1	Activated
Retrograde endocannabinoid signaling	hsa04723	100	31	0.554383971	-1.945667065	0.404	0.559085264	0.871568658	1	Inhibited
Amphetamine addiction	hsa05031	64	17	0.824910841	6.282118118	0.281	0.570663786	0.871568658	1	Activated
Type II diabetes mellitus	hsa04930	45	16	0.313328502	2.665775566	0.754	0.577126342	0.871568658	1	Activated
TGF-beta signaling pathway	hsa04350	76	26	0.323849084	4.351253776	0.73	0.577357349	0.871568658	1	Activated
Endometrial cancer	hsa05213	51	10	0.977799839	10.17505554	0.246	0.583276195	0.871568658	1	Activated
Leukocyte transendothelial migration	hsa04670	106	33	0.542119927	-13.71574261	0.447	0.585818441	0.871568658	1	Inhibited
Wnt signaling pathway	hsa04310	144	33	0.98921127	11.81033912	0.245	0.585859764	0.871568658	1	Activated
Acute myeloid leukemia	hsa05221	52	16	0.57831334	-5.853052477	0.424	0.589879826	0.871568658	1	Inhibited
Morphine addiction	hsa05032	90	28	0.546486962	3.773035349	0.451	0.591648797	0.871568658	1	Activated
Long-term depression	hsa04730	64	17	0.824910841	-11.42801281	0.319	0.614458831	0.88781754	1	Inhibited
Colorectal cancer	hsa05210	61	20	0.441340979	-2.714400108	0.617	0.6265313	0.88781754	1	Inhibited
Chagas disease (American trypanosomiasis)	hsa05142	94	26	0.802121549	12.11221228	0.34	0.627069452	0.88781754	1	Activated
Parkinson's disease	hsa05012	101	27	0.860109739	6.387065171	0.324	0.634741279	0.88781754	1	Activated
Neurotrophin signaling pathway	hsa04722	115	30	0.902580402	-16.84634514	0.31	0.636175629	0.88781754	1	Inhibited
Influenza A	hsa05164	150	44	0.71730767	9.386587591	0.396	0.64156158	0.88781754	1	Activated
Toxoplasmosis	hsa05145	114	28	0.951514488	9.418112813	0.306	0.650421417	0.891077341	1	Activated
Cholinergic synapse	hsa04725	105	30	0.751766386	11.56298584	0.398	0.660231241	0.895561189	1	Activated
SNARE interactions in vesicular transport	hsa04130	31	10	0.516418832	1.146580984	0.615	0.681872532	0.903426905	1	Activated
Bacterial invasion of epithelial cells	hsa05100	66	22	0.398106422	-4.481628042	0.798	0.681977286	0.903426905	1	Inhibited
Measles	hsa05162	121	31	0.925214792	8.941590528	0.347	0.685813125	0.903426905	1	Activated
GnRH signaling pathway	hsa04912	92	22	0.950707772	15.06420568	0.362	0.711253748	0.926204038	1	Activated
Non-small cell lung cancer	hsa05223	53	13	0.886362089	11.41615569	0.394	0.716625022	0.926204038	1	Activated
Regulation of autophagy	hsa04140	27	6	0.890917801	-2.666081371	0.4	0.724064521	0.927073265	1	Inhibited
Hepatitis C	hsa05160	115	31	0.861614487	7.035783276	0.43	0.7383644	0.936628915	1	Activated
RIG-I-like receptor signaling pathway	hsa04622	63	18	0.717146801	4.012448418	0.528	0.7463767	0.938106495	1	Activated
Bile secretion	hsa04976	68	17	0.893634799	1.854583538	0.432	0.753488127	0.938435213	1	Activated
Phosphatidylinositol signaling system	hsa04070	77	20	0.868335016	0.856440272	0.456	0.762794718	0.93930583	1	Activated
Melanogenesis	hsa04916	96	24	0.925144844	-22.64405529	0.434	0.767899657	0.93930583	1	Inhibited
GABAergic synapse	hsa04727	84	26	0.56011949	-1.158045278	0.746	0.782478849	0.948669047	1	Inhibited
Insulin signaling pathway	hsa04910	130	39	0.647857617	11.41669623	0.688	0.805895313	0.963633865	1	Activated
Regulation of actin cytoskeleton	hsa04810	198	61	0.57361953	5.463158088	0.796	0.814551347	0.963633865	1	Activated
Staphylococcus aureus infection	hsa05150	38	9	0.882248366	4.728365048	0.526	0.820340113	0.963633865	1	Activated
Serotonergic synapse	hsa04726	110	35	0.479475028	-0.257748745	0.975	0.822957389	0.963633865	1	Inhibited
Jak-STAT signaling pathway	hsa04630	139	35	0.950547049	-2.547197298	0.511	0.836476451	0.968081429	1	Inhibited
Osteoclast differentiation	hsa04380	112	34	0.611052033	-3.094818436	0.815	0.845188454	0.968081429	1	Inhibited
Olfactory transduction	hsa04740	92	11	0.999996088	6.080371936	0.502	0.847954537	0.968081429	1	Activated
Dopaminergic synapse	hsa04728	123	35	0.773380397	2.504563514	0.687	0.867317032	0.972068311	1	Activated
Carbohydrate digestion and absorption	hsa04973	36	10	0.728326395	0.837724589	0.761	0.881338548	0.972068311	1	Activated
Epithelial cell signaling in Helicobacter pylori infection	hsa05120	63	19	0.617109354	-0.644782082	0.924	0.890525252	0.972068311	1	Inhibited
Pancreatic secretion	hsa04972	88	27	0.581297076	0	1	0.896646865	0.972068311	1	Inhibited
Phototransduction	hsa04744	27	5	0.954714588	2.269542518	0.614	0.89928325	0.972068311	1	Activated
Legionellosis	hsa05134	50	14	0.735268828	1.475594454	0.802	0.901137347	0.972068311	1	Activated
Alzheimer's disease	hsa05010	140	39	0.827918524	-2.216793088	0.724	0.906195238	0.972068311	1	Inhibited
Hedgehog signaling pathway	hsa04340	54	13	0.901905453	-2.789816339	0.669	0.908209809	0.972068311	1	Inhibited
Amoebiasis	hsa05146	99	29	0.694196934	-0.550565362	0.921	0.925335734	0.982720896	1	Inhibited
Long-term potentiation	hsa04720	64	14	0.963367544	5.53116584	0.692	0.936970119	0.986994814	1	Activated
Huntington's disease	hsa05016	145	41	0.801874528	0.686978716	0.863	0.946779695	0.986994814	1	Activated
Notch signaling pathway	hsa04330	44	11	0.853999061	-2.048074108	0.862	0.961647621	0.986994814	1	Inhibited
Glutamatergic synapse	hsa04724	116	31	0.874407011	-1.302194516	0.845	0.962477844	0.986994814	1	Inhibited
Taste transduction	hsa04742	36	8	0.91414399	-1.266640584	0.819	0.965381789	0.986994814	1	Inhibited
Vasopressin-regulated water reabsorption	hsa04962	38	8	0.941170496	-0.508192527	0.873	0.983053952	0.997561599	1	Inhibited
Cocaine addiction	hsa05030	48	11	0.922018338	-0.104189843	0.985	0.995650028	0.997561599	1	Inhibited
Asthma	hsa05310	21	4	0.930983517	0	1	0.997561599	0.997561599	1	Inhibited

SPIA analysis result from TCGA cohort

Name	ID	pSize	NDE	pNDE	tA	pPERT	pG	pGFdr	pGFWER	Status
Focal adhesion	hsa04510	191	108	9.63E-003	-1.91E+001	0.000005	8.60E-007	1.11E-004	1.18E-004	Inhibited
Pathways in cancer	hsa05200	306	165	1.88E-002	-1.83E+001	0.000005	1.61E-006	1.11E-004	2.21E-004	Inhibited
Gap junction	hsa04540	85	48	6.91E-002	-9.88E+000	0.000005	5.49E-006	2.51E-004	7.52E-004	Inhibited
Prostate cancer	hsa05215	81	44	1.46E-001	-9.40E+000	0.000005	1.10E-005	3.78E-004	1.51E-003	Inhibited
RNA transport	hsa03013	129	88	2.22E-006	-5.06E-002	0.852	2.68E-005	7.35E-004	3.68E-003	Inhibited
Cell cycle	hsa04110	115	77	2.65E-005	-1.79E+000	0.439	1.44E-004	3.28E-003	1.97E-002	Inhibited
Oocyte meiosis	hsa04114	99	63	1.11E-003	3.19E+000	0.175	1.86E-003	3.64E-002	2.55E-001	Activated
Melanoma	hsa05218	68	36	2.36E-001	-1.34E+001	0.001	2.21E-003	3.79E-002	3.03E-001	Inhibited
Fanconi anemia pathway	hsa03460	47	33	1.59E-003	4.23E-001	0.361	4.87E-003	7.41E-002	6.67E-001	Activated
ECM-receptor interaction	hsa04512	78	45	5.19E-002	-2.58E+000	0.014	5.97E-003	8.18E-002	8.18E-001	Inhibited
Aldosterone-regulated sodium reabsorption	hsa04960	36	18	4.64E-001	-2.09E+000	0.003	1.05E-002	1.31E-001	1.00E+000	Inhibited
Small cell lung cancer	hsa05222	78	43	1.21E-001	-5.51E+000	0.016	1.40E-002	1.60E-001	1.00E+000	Inhibited
Adipocytokine signaling pathway	hsa04920	65	30	6.55E-001	-3.14E+000	0.004	1.82E-002	1.92E-001	1.00E+000	Inhibited
Olfactory transduction	hsa04740	92	45	4.61E-001	5.53E+000	0.007	2.17E-002	2.13E-001	1.00E+000	Activated
Bladder cancer	hsa05219	41	26	3.27E-002	1.83E+000	0.149	3.08E-002	2.82E-001	1.00E+000	Activated
T cell receptor signaling pathway	hsa04660	102	50	4.46E-001	5.97E+000	0.013	3.57E-002	2.99E-001	1.00E+000	Activated
Herpes simplex infection	hsa05168	155	73	6.08E-001	4.49E+000	0.01	3.71E-002	2.99E-001	1.00E+000	Activated
Insulin signaling pathway	hsa04910	130	62	5.51E-001	9.53E+000	0.013	4.25E-002	3.24E-001	1.00E+000	Activated
Progesterone-mediated oocyte maturation	hsa04914	80	49	1.09E-002	3.06E-001	0.802	5.03E-002	3.63E-001	1.00E+000	Activated
Endometrial cancer	hsa05213	51	30	7.66E-002	2.44E+000	0.197	7.84E-002	5.37E-001	1.00E+000	Activated
Non-small cell lung cancer	hsa05223	53	30	1.28E-001	3.50E+000	0.132	8.57E-002	5.59E-001	1.00E+000	Activated
B cell receptor signaling pathway	hsa04662	71	37	2.75E-001	3.41E+000	0.066	9.08E-002	5.66E-001	1.00E+000	Activated
Pancreatic cancer	hsa05212	68	39	7.40E-002	-1.81E+000	0.26	9.52E-002	5.67E-001	1.00E+000	Inhibited
Colorectal cancer	hsa05210	61	33	1.98E-001	-1.32E+000	0.104	1.01E-001	5.75E-001	1.00E+000	Inhibited
Protein processing in endoplasmic reticulum	hsa04141	151	82	6.58E-002	8.52E-001	0.351	1.10E-001	6.04E-001	1.00E+000	Activated
Intestinal immune network for IgA production	hsa04672	37	19	3.97E-001	5.95E-001	0.068	1.24E-001	6.56E-001	1.00E+000	Activated
HTLV-I infection	hsa05166	236	126	5.02E-002	-1.18E+000	0.572	1.31E-001	6.63E-001	1.00E+000	Inhibited
Salivary secretion	hsa04970	79	46	4.14E-002	-1.86E-001	0.807	1.47E-001	7.00E-001	1.00E+000	Inhibited
Regulation of actin cytoskeleton	hsa04810	198	94	5.72E-001	-5.72E+000	0.059	1.48E-001	7.00E-001	1.00E+000	Inhibited
VEGF signaling pathway	hsa04370	68	30	7.71E-001	2.94E+000	0.052	1.69E-001	7.28E-001	1.00E+000	Activated
Calcium signaling pathway	hsa04020	173	78	7.92E-001	-3.74E+000	0.051	1.70E-001	7.28E-001	1.00E+000	Inhibited
Chronic myeloid leukemia	hsa05220	69	37	2.01E-001	1.91E+000	0.201	1.70E-001	7.28E-001	1.00E+000	Activated
Axon guidance	hsa04360	122	62	2.86E-001	-2.27E+000	0.163	1.90E-001	7.69E-001	1.00E+000	Inhibited
Pathogenic Escherichia coli infection	hsa05130	44	21	5.67E-001	2.59E+000	0.083	1.91E-001	7.69E-001	1.00E+000	Activated
Tuberculosis	hsa05152	151	70	6.75E-001	4.88E+000	0.073	1.98E-001	7.74E-001	1.00E+000	Activated
Serotonergic synapse	hsa04726	110	52	5.87E-001	2.00E+000	0.09	2.08E-001	7.92E-001	1.00E+000	Activated
Glioma	hsa05214	61	34	1.35E-001	-1.92E+000	0.41	2.15E-001	7.97E-001	1.00E+000	Inhibited
mTOR signaling pathway	hsa04150	57	33	8.30E-002	2.17E-001	0.71	2.26E-001	8.14E-001	1.00E+000	Activated
Neurotrophin signaling pathway	hsa04722	115	58	3.23E-001	-2.98E+000	0.191	2.33E-001	8.20E-001	1.00E+000	Inhibited
Renal cell carcinoma	hsa05211	63	36	8.85E-002	3.74E-001	0.73	2.42E-001	8.28E-001	1.00E+000	Activated
Thyroid cancer	hsa05216	28	15	3.39E-001	1.01E+000	0.198	2.48E-001	8.29E-001	1.00E+000	Activated
Viral carcinogenesis	hsa05203	171	88	1.93E-001	1.76E-001	0.371	2.60E-001	8.47E-001	1.00E+000	Activated
Circadian rhythm - mammal	hsa04710	21	10	5.94E-001	1.09E+000	0.124	2.66E-001	8.47E-001	1.00E+000	Activated
Hepatitis C	hsa05160	115	57	3.93E-001	1.63E+000	0.21	2.88E-001	8.87E-001	1.00E+000	Activated
Dorso-ventral axis formation	hsa04320	22	13	2.00E-001	-1.19E-001	0.496	3.29E-001	8.87E-001	1.00E+000	Inhibited
Legionellosis	hsa05134	50	25	4.36E-001	-1.03E+000	0.231	3.32E-001	8.87E-001	1.00E+000	Inhibited
Dilated cardiomyopathy	hsa05414	85	45	2.03E-001	2.26E-001	0.499	3.34E-001	8.87E-001	1.00E+000	Activated
Melanogenesis	hsa04916	96	50	2.34E-001	-3.20E+000	0.463	3.49E-001	8.87E-001	1.00E+000	Inhibited
Pertussis	hsa05133	66	28	8.44E-001	2.01E+000	0.132	3.56E-001	8.87E-001	1.00E+000	Activated
Alcoholism	hsa05034	119	51	8.83E-001	3.83E+000	0.127	3.58E-001	8.87E-001	1.00E+000	Activated
Arrhythmogenic right ventricular cardiomyopathy (ARVC)	hsa05412	69	32	6.43E-001	-2.13E-001	0.179	3.64E-001	8.87E-001	1.00E+000	Inhibited
Long-term potentiation	hsa04720	64	34	2.36E-001	1.79E+000	0.491	3.66E-001	8.87E-001	1.00E+000	Activated
ErbB signaling pathway	hsa04012	83	40	5.20E-001	3.27E+000	0.226	3.69E-001	8.87E-001	1.00E+000	Activated
Graft-versus-host disease	hsa05332	28	11	8.64E-001	2.53E-001	0.139	3.75E-001	8.87E-001	1.00E+000	Activated
Wnt signaling pathway	hsa04310	144	69	5.28E-001	1.87E+000	0.228	3.75E-001	8.87E-001	1.00E+000	Activated
Type I diabetes mellitus	hsa04940	34	15	7.28E-001	2.53E-001	0.167	3.78E-001	8.87E-001	1.00E+000	Activated
Antigen processing and presentation	hsa04612	49	21	8.01E-001	6.29E-001	0.154	3.82E-001	8.87E-001	1.00E+000	Activated
Allograft rejection	hsa05330	29	11	8.97E-001	2.53E-001	0.138	3.82E-001	8.87E-001	1.00E+000	Activated
Mineral absorption	hsa04978	46	23	4.43E-001	-1.06E-001	0.284	3.87E-001	8.87E-001	1.00E+000	Inhibited
Parkinson's disease	hsa05012	101	52	2.64E-001	6.50E-001	0.496	3.97E-001	8.87E-001	1.00E+000	Activated
Salmonella infection	hsa05132	74	39	2.36E-001	6.67E-001	0.56	4.00E-001	8.87E-001	1.00E+000	Activated
Acute myeloid leukemia	hsa05221	52	28	2.34E-001	-5.63E-001	0.579	4.06E-001	8.87E-001	1.00E+000	Inhibited
Bile secretion	hsa04976	68	36	2.36E-001	2.06E-001	0.601	4.19E-001	8.87E-001	1.00E+000	Activated
Amyotrophic lateral sclerosis (ALS)	hsa05014	48	25	3.29E-001	7.23E-001	0.453	4.33E-001	8.87E-001	1.00E+000	Activated
Amoebiasis	hsa05146	99	52	2.03E-001	-2.55E-001	0.74	4.36E-001	8.87E-001	1.00E+000	Inhibited
Endocrine and other factor-regulated calcium reabsorption	hsa04961	46	24	3.31E-001	-6.26E-001	0.461	4.39E-001	8.87E-001	1.00E+000	Inhibited
Autoimmune thyroid disease	hsa05320	39	14	9.52E-001	2.53E-001	0.162	4.43E-001	8.87E-001	1.00E+000	Activated
Tight junction	hsa04530	124	52	9.22E-001	1.28E+000	0.171	4.49E-001	8.87E-001	1.00E+000	Activated
Maturity onset diabetes of the young	hsa04950	21	7	9.41E-001	6.93E-001	0.168	4.50E-001	8.87E-001	1.00E+000	Activated
Fc epsilon RI signaling pathway	hsa04664	71	29	9.05E-001	2.64E+000	0.177	4.53E-001	8.87E-001	1.00E+000	Activated
Amphetamine addiction	hsa05031	64	25	9.39E-001	1.13E+000	0.18	4.69E-001	9.04E-001	1.00E+000	Activated
Gastric acid secretion	hsa04971	71	35	4.51E-001	6.49E-001	0.397	4.87E-001	9.04E-001	1.00E+000	Activated
p53 signaling pathway	hsa04115	64	32	4.14E-001	5.39E-001	0.434	4.88E-001	9.04E-001	1.00E+000	Activated
Rheumatoid arthritis	hsa05323	76	31	9.12E-001	3.66E-001	0.204	4.99E-001	9.04E-001	1.00E+000	Activated
Leishmaniasis	hsa05140	57	23	8.99E-001	1.21E+000	0.208	5.00E-001	9.04E-001	1.00E+000	Activated
GnRH signaling pathway	hsa04912	92	47	3.03E-001	1.31E+000	0.619	5.02E-001	9.04E-001	1.00E+000	Activated
Cocaine addiction	hsa05030	48	14	9.97E-001	-7.51E-001	0.208	5.34E-001	9.18E-001	1.00E+000	Inhibited
Basal cell carcinoma	hsa05217	53	25	5.94E-001	-1.85E+000	0.365	5.48E-001	9.18E-001	1.00E+000	Inhibited
Jak-STAT signaling pathway	hsa04630	139	57	9.57E-001	6.75E-001	0.228	5.50E-001	9.18E-001	1.00E+000	Activated

Viral myocarditis	hsa05416	60	21	9.84E-001	6.47E-001	0.223	5.52E-001	9.18E-001	1.00E+000	Activated
Shigellosis	hsa05131	55	29	2.78E-001	3.31E-001	0.791	5.53E-001	9.18E-001	1.00E+000	Activated
Cholinergic synapse	hsa04725	105	51	4.81E-001	1.52E+000	0.462	5.56E-001	9.18E-001	1.00E+000	Activated
Long-term depression	hsa04730	64	32	4.14E-001	9.49E-001	0.538	5.57E-001	9.18E-001	1.00E+000	Activated
Type II diabetes mellitus	hsa04930	45	17	9.35E-001	1.11E+000	0.245	5.67E-001	9.18E-001	1.00E+000	Activated
Huntington's disease	hsa05016	145	74	2.47E-001	-8.79E-003	0.992	5.90E-001	9.18E-001	1.00E+000	Inhibited
Epstein-Barr virus infection	hsa05169	169	80	5.85E-001	9.81E-001	0.428	5.97E-001	9.18E-001	1.00E+000	Activated
PPAR signaling pathway	hsa03320	66	29	7.77E-001	-2.58E-001	0.324	5.99E-001	9.18E-001	1.00E+000	Inhibited
Staphylococcus aureus infection	hsa05150	38	13	9.69E-001	1.15E+000	0.262	6.02E-001	9.18E-001	1.00E+000	Activated
Bacterial invasion of epithelial cells	hsa05100	66	34	3.19E-001	-4.69E-001	0.833	6.18E-001	9.18E-001	1.00E+000	Inhibited
Toxoplasmosis	hsa05145	114	52	7.18E-001	1.28E+000	0.373	6.21E-001	9.18E-001	1.00E+000	Activated
Transcriptional misregulation in cancer	hsa05202	152	77	2.71E-001	0.00E+000	1	6.25E-001	9.18E-001	1.00E+000	Inhibited
Vascular smooth muscle contraction	hsa04270	106	54	2.95E-001	-2.21E-001	0.927	6.28E-001	9.18E-001	1.00E+000	Inhibited
Chemokine signaling pathway	hsa04062	171	84	4.00E-001	1.22E+000	0.685	6.29E-001	9.18E-001	1.00E+000	Activated
Notch signaling pathway	hsa04330	44	21	5.67E-001	-1.07E+000	0.487	6.31E-001	9.18E-001	1.00E+000	Inhibited
GABAergic synapse	hsa04727	84	43	3.08E-001	3.88E-002	0.933	6.45E-001	9.18E-001	1.00E+000	Activated
Vibrio cholerae infection	hsa05110	49	20	8.72E-001	3.20E-001	0.331	6.47E-001	9.18E-001	1.00E+000	Activated
Pancreatic secretion	hsa04972	88	44	3.84E-001	9.50E-002	0.758	6.50E-001	9.18E-001	1.00E+000	Activated
Lysosome	hsa04142	109	49	7.60E-001	-4.40E-002	0.391	6.58E-001	9.18E-001	1.00E+000	Inhibited
Alzheimer's disease	hsa05010	140	70	3.36E-001	1.22E-001	0.897	6.63E-001	9.18E-001	1.00E+000	Activated
RNA degradation	hsa03018	66	31	6.06E-001	1.57E-001	0.511	6.72E-001	9.19E-001	1.00E+000	Activated
Complement and coagulation cascades	hsa04610	64	27	8.50E-001	2.15E+000	0.371	6.79E-001	9.19E-001	1.00E+000	Activated
Natural killer cell mediated cytotoxicity	hsa04650	109	42	9.80E-001	3.92E+000	0.332	6.91E-001	9.19E-001	1.00E+000	Activated
Cytosolic DNA-sensing pathway	hsa04623	48	22	6.65E-001	2.45E-001	0.49	6.91E-001	9.19E-001	1.00E+000	Activated
Phototransduction	hsa04744	27	8	9.83E-001	6.25E-001	0.349	7.10E-001	9.36E-001	1.00E+000	Activated
Influenza A	hsa05164	150	61	9.69E-001	1.21E+000	0.366	7.22E-001	9.42E-001	1.00E+000	Activated
Vasopressin-regulated water reabsorption	hsa04962	38	19	4.59E-001	1.25E-001	0.82	7.44E-001	9.52E-001	1.00E+000	Activated
Regulation of autophagy	hsa04140	27	11	8.25E-001	-3.71E-001	0.467	7.53E-001	9.52E-001	1.00E+000	Inhibited
MAPK signaling pathway	hsa04010	252	120	5.57E-001	7.44E-001	0.693	7.53E-001	9.52E-001	1.00E+000	Activated
Measles	hsa05162	121	50	9.38E-001	1.15E+000	0.423	7.64E-001	9.52E-001	1.00E+000	Activated
Toll-like receptor signaling pathway	hsa04620	88	33	9.81E-001	1.27E+000	0.419	7.76E-001	9.52E-001	1.00E+000	Activated
Morphine addiction	hsa05032	90	40	7.75E-001	4.34E-001	0.541	7.84E-001	9.52E-001	1.00E+000	Activated
Sulfur relay system	hsa04122	9	4	7.02E-001	-1.25E-001	0.603	7.87E-001	9.52E-001	1.00E+000	Inhibited
Carbohydrate digestion and absorption	hsa04973	36	13	9.44E-001	1.72E-001	0.458	7.95E-001	9.52E-001	1.00E+000	Activated
Systemic lupus erythematosus	hsa05322	68	33	5.04E-001	-7.80E-002	0.866	7.99E-001	9.52E-001	1.00E+000	Inhibited
Phosphatidylinositol signaling system	hsa04070	77	38	4.41E-001	0.00E+000	1	8.02E-001	9.52E-001	1.00E+000	Inhibited
Retrograde endocannabinoid signaling	hsa04723	100	48	5.29E-001	-5.29E-002	0.86	8.13E-001	9.52E-001	1.00E+000	Inhibited
Dopaminergic synapse	hsa04728	123	49	9.70E-001	5.91E-001	0.476	8.19E-001	9.52E-001	1.00E+000	Activated
Taste transduction	hsa04742	36	15	8.19E-001	4.59E-001	0.566	8.20E-001	9.52E-001	1.00E+000	Activated
Hedgehog signaling pathway	hsa04340	54	23	8.19E-001	-5.06E-001	0.59	8.35E-001	9.61E-001	1.00E+000	Inhibited
Leukocyte transendothelial migration	hsa04670	106	46	8.47E-001	-1.21E+000	0.612	8.59E-001	9.75E-001	1.00E+000	Inhibited
Fc gamma R-mediated phagocytosis	hsa04666	83	37	7.61E-001	-7.47E-001	0.699	8.68E-001	9.75E-001	1.00E+000	Inhibited
Osteoclast differentiation	hsa04380	112	51	7.22E-001	5.65E-001	0.761	8.79E-001	9.75E-001	1.00E+000	Activated
Chagas disease (American trypanosomiasis)	hsa05142	94	41	8.24E-001	7.40E-001	0.669	8.80E-001	9.75E-001	1.00E+000	Activated
Epithelial cell signaling in Helicobacter pylori infection	hsa05120	63	27	8.22E-001	3.65E-001	0.677	8.83E-001	9.75E-001	1.00E+000	Activated
NF-kappa B signaling pathway	hsa04064	83	36	8.24E-001	5.52E-001	0.701	8.95E-001	9.81E-001	1.00E+000	Activated
Apoptosis	hsa04210	82	38	6.51E-001	2.11E-001	0.925	9.07E-001	9.81E-001	1.00E+000	Activated
Prion diseases	hsa05020	35	16	6.63E-001	5.94E-002	0.933	9.16E-001	9.81E-001	1.00E+000	Activated
Neuroactive ligand-receptor interaction	hsa04080	253	101	9.96E-001	-1.95E-001	0.624	9.17E-001	9.81E-001	1.00E+000	Inhibited
TGF-beta signaling pathway	hsa04350	76	32	8.69E-001	-2.57E-001	0.835	9.58E-001	9.96E-001	1.00E+000	Inhibited
Glutamatergic synapse	hsa04724	116	52	7.74E-001	-2.09E-002	0.983	9.69E-001	9.96E-001	1.00E+000	Inhibited
African trypanosomiasis	hsa05143	31	13	8.00E-001	-8.86E-003	0.964	9.71E-001	9.96E-001	1.00E+000	Inhibited
NOD-like receptor signaling pathway	hsa04621	54	21	9.28E-001	2.05E-001	0.837	9.73E-001	9.96E-001	1.00E+000	Activated
SNARE interactions in vesicular transport	hsa04130	31	11	9.42E-001	-5.83E-002	0.861	9.81E-001	9.96E-001	1.00E+000	Inhibited
RIG-I-like receptor signaling pathway	hsa04622	63	25	9.24E-001	-8.32E-002	0.911	9.87E-001	9.96E-001	1.00E+000	Inhibited
Asthma	hsa05310	21	8	8.68E-001	0.00E+000	1	9.91E-001	9.96E-001	1.00E+000	Inhibited
Cytokine-cytokine receptor interaction	hsa04060	233	100	9.44E-001	-8.32E-002	0.934	9.93E-001	9.96E-001	1.00E+000	Inhibited
Malaria	hsa05144	44	17	9.17E-001	0.00E+000	1	9.96E-001	9.96E-001	1.00E+000	Inhibited
